# 3-Nitroatenolol: First Synthesis, Chiral Resolution and Enantiomers’ Absolute Configuration

**DOI:** 10.3390/molecules29071598

**Published:** 2024-04-03

**Authors:** Rosa Sparaco, Pierfrancesco Cinque, Antonia Scognamiglio, Angela Corvino, Giuseppe Caliendo, Ferdinando Fiorino, Elisa Magli, Elisa Perissutti, Vincenzo Santagada, Beatrice Severino, Paolo Luciano, Marcello Casertano, Anna Aiello, Gustavo Yuri Martins Viegas, Gilberto De Nucci, Francesco Frecentese

**Affiliations:** 1 Department of Pharmacy, University of Naples Federico II, Via D. Montesano 49, 80131 Naples, Italy; rosa.sparaco@unina.it (R.S.); pierfrancesco.cinque@unina.it (P.C.); antonia.scognamiglio@unina.it (A.S.); angela.corvino@unina.it (A.C.); caliendo@unina.it (G.C.); fefiorin@unina.it (F.F.); perissut@unina.it (E.P.); santagad@unina.it (V.S.); bseverin@unina.it (B.S.); paolo.luciano@unina.it (P.L.); marcello.casertano@unina.it (M.C.); anna.aiello@unina.it (A.A.); frecente@unina.it (F.F.); 2 Department of Public Health, University of Naples Federico II, Via Pansini 5, 80131 Naples, Italy; elisa.magli@unina.it; 3 Department of Pharmacology, Faculty of Medical Sciences, State University of Campinas (UNICAMP), Campinas 13083-887, SP, Brazil; g272640@dac.unicamp.br; 4 Department of Health Studies, Metropolitan University of Santos (UNIMES), Santos 11045-002, SP, Brazil

**Keywords:** 3-nitroatenolol, chiral HPLC, NMR absolute configuration assignment, Riguera’s method

## Abstract

4-Nitro and 7-nitro propranolol have been recently synthesized and characterized by us. (±)-4-NO_2_-propranolol has been shown to act as a selective antagonist of 6-nitrodopamine (6-ND) receptors in the right atrium of rats. As part of our follow-up to this study, herein, we describe the first synthesis of (±)-3-nitroatenolol as a probe to evaluate the potential nitration of atenolol by endothelium. Chiral chromatography was used to produce pure enantiomers. By using Riguera’s method, which is based on the sign distribution of ΔδH, the absolute configuration of the secondary alcohol was determined.

## 1. Introduction

β-blockers are a commonly prescribed medication for cardiovascular conditions such as heart failure [1] and to prevent major adverse cardiac events following myocardial infarction [2]. The use of β-blockers for the treatment of angina pectoris was pioneered by Scottish pharmacologist Sir James Black in 1958, for which he was awarded the Nobel Prize [3]. FDA approved the cardioselective β-blocker atenolol, also known as 4-(2-hydroxy-3-isopropyl-aminopropoxy) phenylacetamide, in September 1981 [4]. It was developed by Alvogen Malta and sold under the brand name Tenormin^®^.

6-Nitrodopamine (6-ND) has been considered a primary endogenous modulator of chronotropism in the isolated right atrium of rats [5]. It enhances the chronotropic effects of the classical catecholamines and is 10,000 times more potent than dopamine. It is 100 times more potent than noradrenaline and adrenaline [6]. Owing to the critical significance of endothelium-derived 6-ND, a great deal of research has been conducted to characterize its effects on the cardiovascular system and ascertain the amounts released from cardiac tissues [7].

To evaluate the possibility of endothelium-mediated propranolol nitration, we have recently synthesized and characterized two nitro derivatives of propranolol, namely 4-nitro and 7-nitro propranolol. Chiral HPLC was used to separate the enantiomers with very high yields and excellent enantiopurity. Lastly, we employed double derivatization with MPA and NMR investigations in accordance with Riguera’s method to determine the enantiomers’ absolute configuration [8].

The effects of propranolol, 4-NO_2_-propranolol, 7-NO_2_-propranolol and their respective enantiomers were investigated in the isolated right atrium of rats. The results clearly demonstrate that (±)-4-NO_2_-propranolol causes a reduction in atrial rate at concentrations that do not affect the positive chronotropic effect induced by noradrenaline and adrenaline but do inhibit the positive chronotropic effect induced by 6-ND. The finding that the enantiomer (*S*)-(+)-4-NO_2_-propranolol caused a reduction in the atrial rate, whereas the *(R*)-(−)-4-NO_2_-propranolol was far less potent, indicates that the interaction of 6-ND with its receptor is highly stereoselective [9].

In light of our important results, consisting of the identification of the first selective antagonist of the 6-ND receptor, our interest in the search for new nitro derivatives of β-blockers has become even stronger. Propranolol, a non-selective β-blocker [10], was selected as a historical interest compound in the β-blocker class. The selective action of second-generation β1-blockers is directed towards β1 receptors located in the heart [11]. With a strong affinity for the β1-adrenergic receptor, atenolol is one of the medications from the latter group that is still frequently used in pharmacotherapy today [12,13]. It is frequently used to prevent cardiovascular events and delay the onset of cardiovascular disease [14,15].

To evaluate the potential nitration of atenolol by endothelium, we designed and reported in this paper the first synthesis of (±)-3-nitroatenolol and its pure enantiomers. This is a continuation of our research and an analogy to what has already been studied and published for nitropropranolol derivatives. Chiral chromatography was used to obtain pure enantiomers, and Riguera’s method—which relies on the sign distribution of ΔδH—was used to determine the secondary alcohol’s absolute configuration [16,17].

## 2. Results

**Scheme 1:**
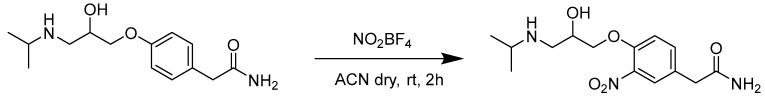
Synthetic procedure for the synthesis of (±)-3-nitroatenolol.

(±)-3-Nitroatenolol was obtained following the procedure described in Scheme 1.

The synthetic procedure consists of the direct nitration of (±)-atenolol by a reaction with a slight excess of nitronium tetrafluoroborate (1.1 eq.) in anhydrous acetonitrile (ACN) at room temperature (rt). Purification was carried out by reversed-phase flash chromatography, using H_2_O + 0.1% TFA and ACN + 0.1% TFA as mobile phases (gradient and relative ratios are reported in Materials and Methods).

**Figure 1:**
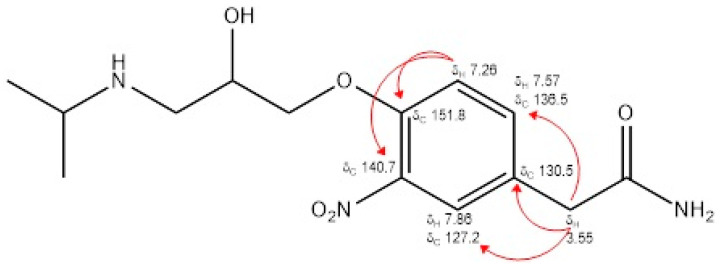
Main NMR signals and correlations of 3-nitroatenolol.

By analyzing the data provided by the ^1^H NMR spectrum and the ^13^C NMR spectrum, interpreted through the analysis of the two-dimensional HSQC and HMBC spectra (Figures S5–S8, respectively), it was possible to assign the position of the nitro group on the aromatic ring of atenolol at the ortho position with respect to the oxy-propanolamine chain. In particular, an analysis of the correlations present in the HMBC spectrum, with reference to those between the singlet at δ 3.55 and the signals of the carbons at δ 127.2, 130.5 and 136.5, and between the doublet at δ 7.26 and the signals of the carbons at δ 130.5, 140.7 and 151.8, made it possible to unambiguously establish the position of the nitro group (Figure 1).

**Figure 2:**
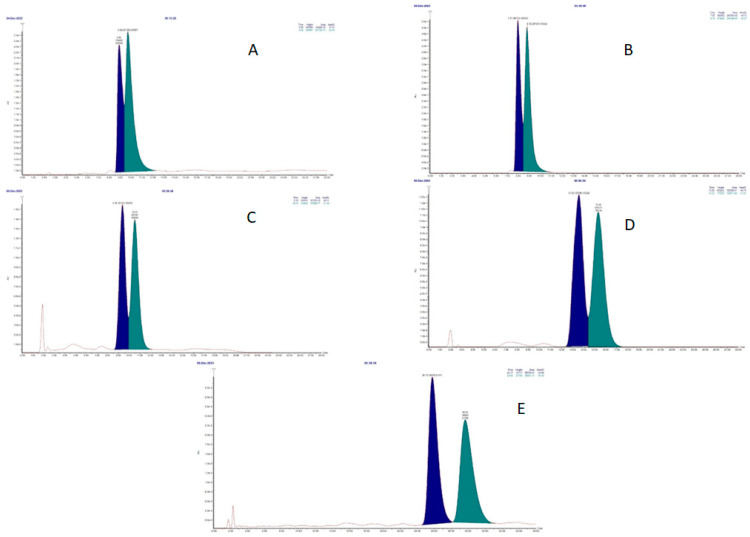
Chiral HPLC chromatograms of (±)-3-nitroatenolol: n-hexane-isopropanol 86:14 *v*/*v* +0.1% DEA (**A**); n-hexane-ethanol 70:30 *v*/*v* +0.1% DEA (**B**); n-hexane-ethanol 75:25 *v*/*v* +0.1% DEA (**C**); n-hexane-ethanol 80:20 *v*/*v* +0.1% DEA (**D**); n-hexane-ethanol 80:20 *v*/*v* (**E**).

The two enantiomers were then separated by chiral HPLC. The development of the chromatographic method started from the conditions used for (±)-4-nitro and (±)-7-nitro propranolol derivatives adopted in our previous study [8], with the same chiral column and with a mobile phase composed as follows: n-hexane-isopropanol 86:14 *v*/*v* + 0.1% diethylamine (DEA). However, the chromatogram showed poor resolution for (±)-3-nitroatenolol under these conditions (Figure 2A).

Subsequent modifications were conducted by replacing isopropanol with ethanol in decreasing concentrations from 30% to 20% and leaving the amount of DEA (0.1%) unchanged. These procedures resulted in a slight improvement in chromatographic resolution, but without allowing the full separation of enantiomer peaks (Figure 2B–D).

A satisfying peak separation to ensure the isolation of pure enantiomers was achieved by the elimination of diethylamine in the mobile phase (n-hexane-ethanol 80:20 *v*/*v*), thus obtaining the chromatogram reported in Figure 2E.

Each enantiomer was further analyzed to determine the purity, which was generally >95% with an enantiomeric excess >95%.

The purity of (+) and (−) enantiomers were assessed by chiral HPLC with the same solvents and conditions as for preparative purification but with a flow rate of 1 mL/min. Chromatograms obtained for each single enantiomer are reported in the Supplementary material (Figures S1 and S2).

The optical rotation value for each enantiomer was determined by optical polar metric analysis. The compound corresponding to the peak with a lower retention time (Figure 2E, t_R_ = 25.77) gave a positive [α]_D_ value; the compound corresponding to peak 2 (Figure 2E, t_R_ = 29.65) gave a negative [α]_D_ value.

Finally, the absolute configuration (AC) of the synthesized and purified compounds was determined by the double derivatization of each 3-nitroatenolol enantiomer with α-methoxyphenylacetic acid (MPA), a specific chiral derivatizing agent followed by NMR analysis of the resulting derivatives.

Researchers working on bioactive chiral compounds have always turned their interest to methods for determining the absolute stereochemistry of individual enantiomers. This is essential to justify the possible different biological activities that these compounds, in many cases, manifest, if the specific receptor area with which they interact can differentiate their biological responses. Among the most versatile, widespread and reliable methods, the literature indicates for assigning absolute configuration to chiral secondary alcohols by NMR the Mosher’s method, as further optimized by Professor Ricardo Riguera’s research group at the University of Santiago in Spain [16,17].

(+)-3-Nitroatenolol and (−)-3-nitroatenolol were reacted both with (*R*)-(−)-α-methoxyphenylacetic acid, (*R*)-MPA, and with (*S*)-(+)-α-methoxyphenylacetic acid, (*S*)-MPA. Each enantiomer was separately derivatized in dichloromethane (DCM) and using the coupling reaction agent 1-ethyl-3-(3-dimethylaminopropyl)carbodiimide (EDC) in the presence of a catalytic amount of the base 4-dimethylaminopyridine (DMAP). After solvent evaporation, the MPA-esters were obtained in pure form by reversed-phase (RP)-HPLC.

**Scheme 2:**
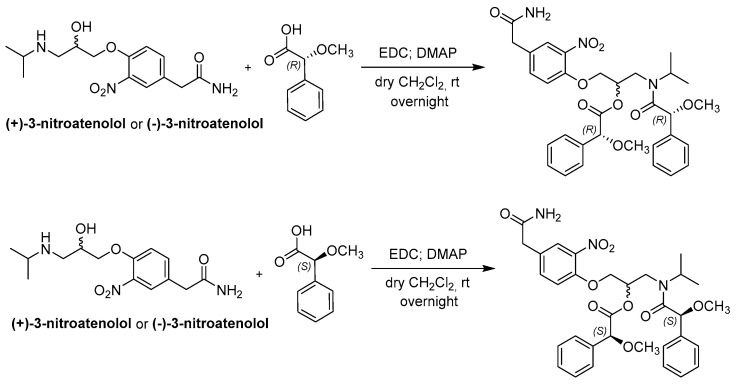
Synthetic procedure for the synthesis of bis-MPA derivatives of atenolol nitro-analogues.

The synthetic procedure is reported in Scheme 2.

**Figure 4:**
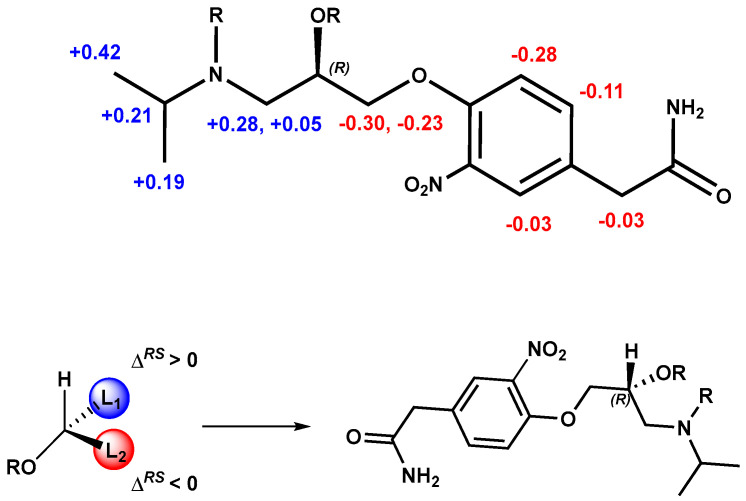
Absolute configuration assignment of (*R*)-(+)- 3-nitroatenolol.

The absolute configuration of the secondary alcohol was assigned considering the sign distribution of Δ*δ*_H_ (calculated as *δ*_H_^R^ − *δ*_H_^S^), obtained from the comparison of the NMR spectra of the different MPA-derived compounds. NMR signals were completely descripted, paying special attention to the protons located at the two substituents of the chiral carbon, which constitute the L1 and L2 groups (Figures 4 and 6). The stereogenic center showed a different and opposite behavior to the shielding/deshielding anisotropic effects exerted by the MPA units around when (*R*)- or (*S*)-MPA were used. The analysis of Δ*δ*^RS^ (*δ*^R^ − *δ*^S^) parameters allowed us to deduce the following:

- The *R* configuration for (+)-3-nitroatenolol;
- The *S* configuration for (−)-3-nitroatenolol.

## 3. Materials and Methods

### 3.1. Chemistry

All commercial reagents and solvents were purchased from Merck (Darmstadt, Germany), TCI Europe (Zwijndrecht, Belgium) and Enamine (Kyiv, Ukraine). Reactions were stirred at 400 rpm by a Heidolph MR Hei-Standard magnetic stirrer. Solutions were concentrated with a Buchi R-114 rotary evaporator at low pressure. All reactions were followed by TLC carried out on Merck silica gel 60 F254 plates with a fluorescent indicator on the plates and were visualized with UV light (254 nm). Flash chromatography was performed using a Biotage Selekt instrument (Biotage, Uppsala, Sweden) equipped with a Biotage Sfar C18 D column. Two mobile phases were used, mobile phase A: H_2_O + 0.1% TFA and mobile phase B: acetonitrile + 0.1% TFA, and they were employed to run gradient conditions. Chiral HPLC resolution was performed using a WATERS Quaternary Gradient Mobile 2535 instrument equipped with WATERS UV/Visible Detector 2489 set to a dual-wavelength UV detection at 254 and 280 nm. The chiral resolutions were achieved on the Kromasil 5-Amycoat column (Phenomenex, 150 mm × 21.2 mm, 5 μm particle size).

LC-MS analysis was performed using a VANQUISH FLEX module comprising a quaternary pump with a degasser, an autosampler, a column oven (set at 40 °C), a diode array detector DAD and a column as specified in the respective methods below. The MS detector (ISQ Thermo Fisher Scientific, Waltham, MA, USA) was configured with an electrospray ionization source. Mass spectra were acquired by scanning from 100 to 700 in 0.2 s. The capillary needle voltage was 3 kV in positive and 2 kV in negative ionization modes, and the source temperature was maintained at 250 °C. Nitrogen was used as the nebulizer gas. Reversed-phase (RP) UHPLC was carried out on a Luna Omega-C18 column (Phenomenex, 3 µm, 50 × 2.1 mm) with a flow rate of 0.600 mL/min. Two mobile phases were used, mobile phase A: H_2_O (LC-MS grade) with 0.1% formic acid and mobile phase B: acetonitrile (LiChrosolv for LC-MS Merck), and they were employed to run gradient conditions from 15% B for 0.20 min, from 15% to 95% for 1.60 min, 95% B for 0.60 min and 15% B for 0.10 min, and these conditions were held for 1.05 min in order to re-equilibrate the column (total run time: 3.55 min). An injection volume of 0.8 µL was used. Data acquisition was performed with Chromeleon 7.

RP-HPLC was carried out on a Luna 3 µM C18 (150 × 3.0 mm) column (Phenomenex, Torrance, CA, USA) using a Knauer Azura Pump 4.1 S instrument equipped with a Knauer K-2301 RI detector (Lab-Service Analytica, Anzola dell’Emilia, Italy) for the purification of MPA derivatives. Melting points, determined using a Buchi Melting Point B-540 instrument, are uncorrected and represent values obtained on recrystallized or chromatographically purified material. Mass spectra of the final products were performed on a Thermo LTQ Orbitrap XL mass spectrometer (Thermo-Fisher, San Josè, CA, USA) in positive mode. MeOH was used as the solvent for compounds infusion. The ^1^H NMR (700 MHz) analyses were recorded on a Bruker Avance Neo spectrometer (Bruker BioSpin Corporation, Billerica, MA, USA); chemical shifts were reported in ppm and were referenced to the residual solvent signal (CD_3_OD: *δ*_H_ = 3.31, *δ*_C_ = 49.0). NMR spectra are reported in Supplementary Materials (Figures S3–S6). A Jasco P-2000 series polarimeter (Jasco Europe, Cremella, Italy) at the sodium D line was used to measure optical rotations.

(±)-2-(4-(2-hydroxy-3-(isopropylamino)propoxy)-3-nitrophenyl)acetamide (±-3-nitroatenolol).

Nitronium tetrafluoroborate (0.55 g, 4.13 mmol) was added to a solution of atenolol (1.00 g, 3.75 mmol) in dry ACN (10 mL). The mixture was stirred at rt for 2 h under a N_2_ atmosphere. After 2 h, the solvent was removed under vacuo, and the crude was purified by reversed-phase flash chromatography using H_2_O + 0.1% TFA (A) e ACN + 0.1% TFA (B) as the mobile phase. The operational conditions were as follows: 5% B for 2 min, linear gradient of 5–70% B in 18 min and 70% B for 4 min. The purity of compounds was assessed by analytical RP-HPLC, employing the same solvents and a gradient of 5–70% B over 20 min. The compound was obtained as trifluoroacetate salt. Yellow oil (0.475 g, 29%). ^1^H NMR (700 MHz, CD_3_OD): δ 7.86 (d, *J* = 2.1 Hz, 1H), 7.56 (dd, *J* = 8.6, 2.2 Hz, 1H), 7.26 (d, *J* = 8.6 Hz; 1H), 4.30–4.24 (m, 2H), 4.19–4.17 (m, 1H), 3.55 (s, 2H, -CH_2_CO-), 3.50–3.43 (m, 1H, -CH(CH_3_)_2_), 3.33–3.24 (m, 2H, -CH_2_NH-), 1.39 (d, *J* = 2.8 Hz, 3H, -CH_3_), 1.37 (d, *J* = 2.8 Hz, 3H, -CH_3_). ^13^C NMR (175 MHz, CD_3_OD): δ 175.8, 151.9, 140.8 (-CNO_2_), 136.5, 130.6, 127.2, 116.2, 72.5 (-CH_2_O-), 66.4 (-CHOH), 52.2 (-CH(CH_3_)_2_), 48.5 (-CH_2_NH-), 41.5 (-CH_2_CO-), 19.3 (-CH_3_), 18.8 (-CH_3_). HRESI-MS *m*/*z* 312.1566 [M + H]^+^ (calculated for C_14_H_22_N_3_O_5_ 312.1554). Pretreatment of the yellow oil with 10% methanolic ammonia solution gave the corresponding (±)-3-nitroatenolol free base as a yellow powder. m.p. 115.0–116.7 °C.

### 3.2. Chiral Resolution

The resolution of the racemic mixture containing (±)-3-nitroatenolol was preceded by pretreatment aimed at removing trifluoroacetate counterion. The racemic mixture was preconcentrate onto a 1-g SCX cartridge (ThermoScientific Hypersep SCX SPE 1000 mg, CODE: 60108-433). The procedure involves conditioning the cartridge with 100% MeOH. (±)-3-Nitroatenolol was solubilized in MeOH and deposited on the column load. Three volumes (2 mL each) of MeOH were eluted. Then, a 10% methanolic ammonia solution was added (3 volumes, 2 mL each). The racemic mixture of the free bases was collected. LC-MS analysis of the eluate did not detect the presence of TFA. LC-MS spectra of (±)-3-nitroatenolol pre-SCX and post-SCX pretreatment are reported in Figures S7 and S8, respectively.

Chiral purification was performed using the Waters 2535 Quaternary Gradient Mobile equipped with the Waters 2489 UV/Visible Detector set to a dual-wavelength UV detection at 254 and 280 nm. The chiral resolutions were achieved on the Kromasil 5-Amycoat column (Phenomenex, 150 mm × 21.2 mm, 5 μm particle size). Two mobile phases, in isocratic condition, were used. Mobile phase A: n-hexane (Chromasolv, Merck, Darmstadt, Germany); mobile phase B: ethanol (Chromasolv, Merck, Darmstadt, Germany). Ratio of the mobile phases: 80(A): 20(B). The racemic mixture was dissolved in ethanol at a concentration of 100 mg/600 µL, and the injection volume was 200 μL (repeated 3 times); the sample was eluted from the column at a flow rate of 15.0 mL/min at rt (pressure: ≈500 psi). At the end of the racemic resolutions, 23.7 mg of each enantiomer was collected. The purity of (+) and (−) enantiomers was assessed by chiral HPLC using a Kromasil 5-Amycoat column (Phenomenex, 150 mm × 4.6 mm, 5 μm particle size) with the same solvents and conditions as for the preparative purification, but with a flow rate of 1 mL/min.

### 3.3. Determination of Optical Rotation Values

The optical rotation values were measured for each enantiomer of the synthesized (±)-3-nitroatenolol at 589 nm with a Jasco P-2000 polarimeter using a 10 cm microcell. Each sample was dissolved in MeOH, and the obtained values are reported in Table 1.

### 3.4. Absolute Configuration Assignment

The absolute configuration of (±)-3-nitroatenolol enantiomers was assigned by applying Riguera method [16] which is based on the use of chiral derivatizing agents through the double derivatization approach of secondary alcohols. In this work, (+)-3-nitroatenolol and (−)-3-nitroatenolol were treated with both *R* and *S* enantiomers of the α-methoxyphenylacetic acid ((*R*)- and (*S*)- (MPA) and thus converted into bis MPA derivatives.

The synthesis was performed according to our previously developed procedure [8,18]. The derivatization reaction was carried out on an aliquot of each pure synthesized nitro-derivative of atenolol: the enantiomers were separately dissolved in dry dichloromethane (800 µL) and treated with either (*R*)-(−)-α-MPA or (*S*)-(+)-α-MPA (2.2 eq.). To this solution, EDC (2.2 eq.), and a catalytic amount of DMAP were also added, stirring the mixture overnight at rt. After this time, the solvent was evaporated under reduced pressure, and the crude material was purified by RP-HPLC on a Luna 3 µM C18 column (Phenomenex, Torrance, CA, USA) with flow rate of 0.5 mL/min and MeOH/H_2_O 6:4 (*v*/*v*) as the mobile phase, affording the desired products in a pure state. The detailed experimental conditions as well as the yield of isolated products are summarized in Table 2.

The proton and carbon resonances of the bis-(*R*)-MPA of (+)-3-nitroatenolol and bis-(*S*)-MPA of (+)-3-nitroatenolol (Figure 3a and Figure 3b, respectively) were assigned by the analysis of key correlations of the 2D NMR experiments (^1^H-^1^H COSY, ^1^H-^13^C HSQC and ^1^H-^13^C HMBC, Figures S10–S13 and S15–S18, respectively).

Bis-(*R*)-MPA derivative of (+)-3-nitroatenolol: HRESI-MS: *m*/*z* 608.2631 [M+H]^+^ (calcd. for C_32_H_38_N_3_O_9_ 608.2603), Figure S14; bis-(*S*)-MPA derivative of (+)-3-nitroatenolol: HRESI-MS: *m*/*z* 608.2619 [M + H]^+^ (calcd. for C_32_H_38_N_3_O_9_ 608.2603), Figure S19.

The stereochemical assignment of (+)-3-nitroatenolol was afforded by the calculation of Δ*δ*_H_ (calculated as *δ*_H_*^R^*− *δ*_H_*^S^*) and analyzing the Δ*δ*_H_ sign distribution of the substituents of the asymmetric carbon of the substrate according to Riguera’s model [16]. These observed differences in the chemical shifts were interpreted and allowed us to assign the *R* absolute configuration to (+)-3-nitroatenolol (Figure 4).

**Figure 5:**
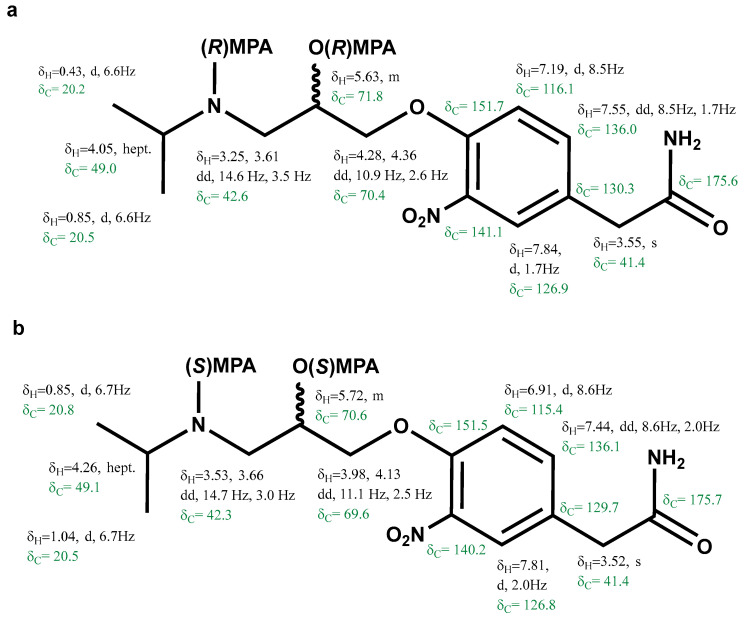
Proton and carbon chemical shifts of the bis-(*R*)-MPA derivative of (−)-3-nitroatenolol (**a**) and bis-(*S*)-MPA derivative of (−)-3-nitroatenolol (**b**).

Analogously, ^1^H and ^13^C chemical shifts were recorded and assigned for the bis-(*R*)-MPA of (−)-3-nitroatenolol and bis-(*S*)-MPA of (−)-3-nitroatenolol, too (Figure 5a and Figure 5b, respectively). ^1^H-NMR and ^1^H-^13^C HMBC are reported in Figures S19 and S20, S22 and S23, respectively. Bis-(*R*)-MPA derivative of (−)-3-nitroatenolol: HRESI-MS: *m*/*z* 608.2595 [M+H]^+^ (calcd. for C_32_H_38_N_3_O_9_ 608.2603), Figure S21; bis-(*S*)-MPA derivative of (−)-3-nitroatenolol: HRESI-MS: *m*/*z* 608.2606 [M+H]^+^ (calcd. for C_32_H_38_N_3_O_9_ 608.2603), Figure S24.

**Figure 6:**
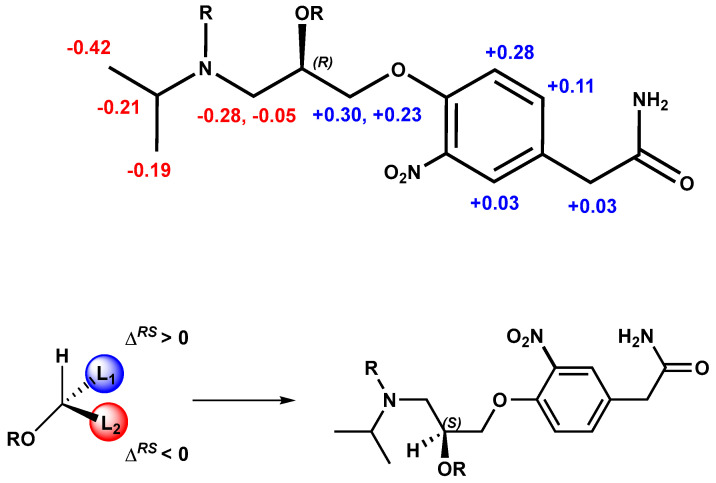
Absolute configuration assignment of (*S*)-(−)- 3-nitroatenolol.

As expected, if the absolute configuration of the alcohol is the opposite, the signs of Δ*δ*_H_^RS^ will also be reversed (Figure 6). Therefore, the *S* absolute configuration can be assigned to the stereogenic center of (−)-3-nitroatenolol.

## 4. Conclusions

The present work has enabled the development of a novel synthetic process for obtaining 3-nitroatenolol. Specifically, the racemic mixture was obtained from the study, and the separation of the enantiomers was conducted by chiral chromatography. Their absolute configuration and optical activity were determined by double derivatization with the chiral derivatizing agent α-methoxyphenylacetic acid, NMR studies and polarimetry.

The racemic mixture and respective enantiomers represent probes to assess the possible nitration of atenolol by endothelium in patients taking the drug.

## Figures and Tables

**Figure 3:**
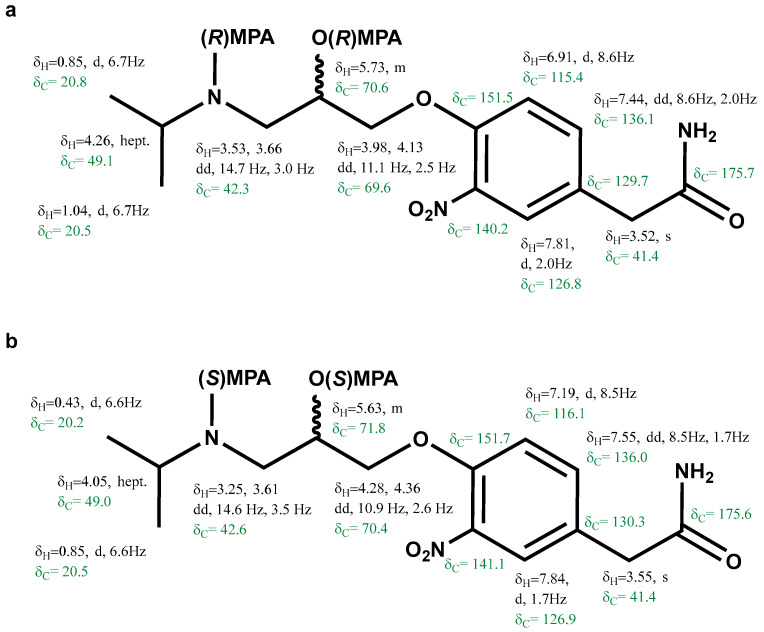
Proton and carbon chemical shifts of the bis-(*R*)-MPA derivative of the (+)-3-nitroatenolol (**a**) and bis-(*S*)-MPA derivative of the (+)-3-nitroatenolol (**b**).

**Table 1:** [α]^20^_D_ values of the atenolol nitro-derivatives.

**Peak 1**	[α]^20^_D_ = +2.88 (c = 0.003, MeOH)	(+)-3-nitroatenolol
**Peak 2**	[α]^20^_D_ = −2.59 (c = 0.002, MeOH)	(−)-3-nitroatenolol

**Table 2:** Experimental conditions for the synthesis of MPA-derived compounds.

Compounds	Reagent (mg)	(*R*)- or (S)-MPA (mg)	EDC (mg)	t_R_ (min.)	Isolated Yield (%)
**bis-(*R*)-MPA derivative of** **(+)-3-nitroatenolol**	2.4	3.0	3.1	13.7	79
**bis-(*S*)-MPA derivative of** **(+)-3-nitroatenolol**	2.3	3.0	3.0	15.9	67
**bis-(*R*)-MPA derivative of** **(−)-3-nitroatenolol**	2.1	3.0	3.0	16.0	69
**bis-(*S*)-MPA derivative of** **(−)-3-nitroatenolol**	2.5	3.0	3.2	13.8	87

## Data Availability

The data presented in this study are available in article and Supplementary Material.

## Supplementary Materials

The following supporting information can be downloaded at: https://www.mdpi.com/article/10.3390/molecules29071598/s1, Figures S1 and S2: Stereochemical purity and LC/MS analyses for 3-nitroatenolol enantiomers; Figures S3–S6: Mono and bidimensional NMR spectra of (±)-3-nitroatenolol; Figures S7 and S8: LC-MS spectra of (±)-3-nitroatenolol pre-SCX and post-SCX pretreatment; Figure S9: HRESI-MS spectrum of (±)-3-nitroatenolol; Figures S10–S19: ^1^H-NMR, COSY, HSQC, HMBC and HRMS spectra for bis-MPA derivatives of (+)-3-nitroatenolol; Figures S20–S25: ^1^H-NMR, HMBC and HRMS spectra for bis-MPA derivatives of (−)-3-nitroatenolol.

## References

[1] Bismpos D., Wintrich J., Hövelmann J., Böhm M. (2024). Latest pharmaceutical approaches across the spectrum of heart failure. Heart Fail. Rev..

[2] Johri N., Mareja P.S., Maurya A., Varshney S., Smritigandha (2023). Role of β-blockers in preventing heart failure and major adverse cardiac events post myocardial infarction. Curr. Cardiol. Rev..

[3] Stapleton M.P. (1997). Sir James Black and propranolol. The role of the basic sciences in the history of cardiovascular pharmacology. Tex. Heart Inst. J..

[4] https://go.drugbank.com/drugs/DB00335.

[5] Britto-Júnior J., Gonçalves de Oliveira M., dos Reis Gati C., Campos R., Moraes M.O., Moraes M.E.A., Mónica F.Z., Antunes E., De Nucci G. (2022). 6-NitroDopamine is an endogenous modulator of rat heart chronotropism. Life Sci..

[6] Britto-Júnior J., Lima A.T., Fuguhara V., Monica F.Z., Antunes E., De Nucci G. (2023). Investigation on the positive chronotropic action of 6-nitrodopamine in the rat isolated atria. Naunyn-Schmiedeberg’s Arch. Pharmacol..

[7] Zatz R., De Nucci G. (2024). Endothelium-Derived Dopamine and 6-Nitrodopamine in the Cardiovascular System. Physiology.

[8] Sparaco R., Scognamiglio A., Corvino A., Caliendo G., Fiorino F., Magli E., Perissutti E., Santagada V., Severino B., Luciano P. (2023). Synthesis, Chiral Resolution and Enantiomers Absolute Configuration of 4-Nitropropranolol and 7-Nitropropranolol. Molecules.

[9] Oliveira D.L., Cardoso V.F., Britto-Júnior J., Fuguhara V., Frecentese F., Sparaco R., Santagada V., Caliendo G., Antunes E., De Nucci G. (2024). The effect of (±)-4-NO_2_-propranolol, (±)-7-NO_2_-propranolol, and (±)-propranolol on the rat isolated right atrium. Naunyn-Schmiedeberg’s Arch. Pharmacol..

[10] Nies A.S., Shand D.G. (1975). Clinical pharmacology of propranolol. Circulation.

[11] Ogrodowczyk M., Dettlaff K., Jelinska A. (2016). Beta-blockerrs: Current state of knowledge and perspectives. Min. Rev. Med. Chem..

[12] Viteri M., Aranibar L. (2023). Oral atenolol compared to oral propranolol for infantile hemangioma. Medwave.

[13] Brodde O.E. (2007). β-adrenoceptor blocker treatment and the cardiac β-adrenoceptor-G-protein(s)-adenylyl cyclase system in chronic heart failure. Naunyn-Schmiedeberg’s Arch. Pharmacol..

[14] Mohan S., Campbell N.R.C. (2008). Hypertension management in Canada: Good news, but important challenges remain. CMAJ.

[15] Gupta A., Whiteley W.N., Godec T., Rostmian S., Ariti C., Mackay J., Whitehouse A., Janani L., Poulter N.R., Sever P.S. (2024). Legacy benefits of blood pressure treatment on cardiovascular events are primarily mediated by improved blood pressure variability: The ASCOT trial. Eur. Heart J..

[16] Seco J.M., Quiñoá E., Riguera R. (2004). The Assignment of Absolute Configuration by NMR. Chem. Rev..

[17] Leiro V., Seco J.M., Quiñoá E., Riguera R. (2008). Cross interaction between auxiliaries: The chirality of amino alcohols by NMR. Org. Lett..

[18] Luciano P., Imperatore C., Senese M., Aiello A., Casertano M., Guo Y.W., Menna M. (2017). Assignment of the Absolute Configuration of Phosphoeleganin via Synthesis of Model Compounds. J. Nat. Prod..

