# Supplementary material for: 3-Nitroatenolol: First Synthesis, Chiral Resolution and Enantiomers’ Absolute Configuration
Source: Molecules. 2024 Apr 3;29(7):1598. doi: 10.3390/molecules29071598 (PMC11013601; doi:10.3390/molecules29071598)
Supplement: Supplementary file 1 [file molecules-29-01598-s001.zip › molecules-2920724-supplementary.pdf]

Supporting Information

3-Nitroatenolol: First Synthesis, Chiral Resolution and Enantiomers' Absolute Configuration

Rosa Sparaco,[1] Pierfrancesco Cinque,[1] Antonia Scognamiglio,[1] Angela Corvino,[1] Giuseppe Caliendo,[1] Ferdinando Fiorino,[1] Elisa Magli,[2] Elisa Perissutti,[1] Vincenzo Santagada,[1] Beatrice Severino,[1] Paolo Luciano,[1] Marcello Casertano,[1] Anna Aiello,[1] Gustavo Yuri Martins Viegas,[3,4] Gilberto De Nucci,[3,4,*] and Francesco Frecentese[1]

[1] *Department of Pharmacy, University of Naples Federico II, Via D. Montesano 49, Naples, Italy*

[2] *Department of Public Health, University of Naples Federico II, Via Pansini 5, Naples, Italy*

[3] *Department of Pharmacology, Faculty of Medical Sciences, State University of Campinas (UNICAMP), São Paulo, Campinas, Brazil*

[4] *Department of Health Studies, Metropolitan University of Santos (UNIMES), Santos-SP, Brazil.*

Content:

Chiral analytical HPLC: stereochemical purity and LC/MS analyses for (+)-3-nitroatenol and (-)-3-nitroatenolol..…………………………..……………………………………..…... pages S2-S5

Mono and bidimensional NMR spectra of (±)-3-nitroatenolol……..……………..………………………………………………….........pages S6-S7

LC-MS spectra of (±)-3-nitroatenolol pre-SCX and post-SCX pretreatment………. ……….page S8

^{1}H-NMR, COSY, HSQC, HMBC and HRMS spectra for bis-MPA derivatives of (+)-3-nitroatenolol…………………………..…………..……………………………… pages S9-S14

^{1}H-NMR, HMBC and HRMS spectra for bis-MPA derivatives of (-)-3-nitroatenolol……………………………..………………………..…………………… pages S15-S18

Peak 1 - *(+)*-3-nitroatenolol

Item	Specification	Result
**Appearance**	Yellow powder	Compliant
**Purity**	LC-MS purity >95%	Compliant
**Enantiomeric purity**	HPLC > 95%	Compliant
**Amount**	23.7 mg	

Stereochemical purity

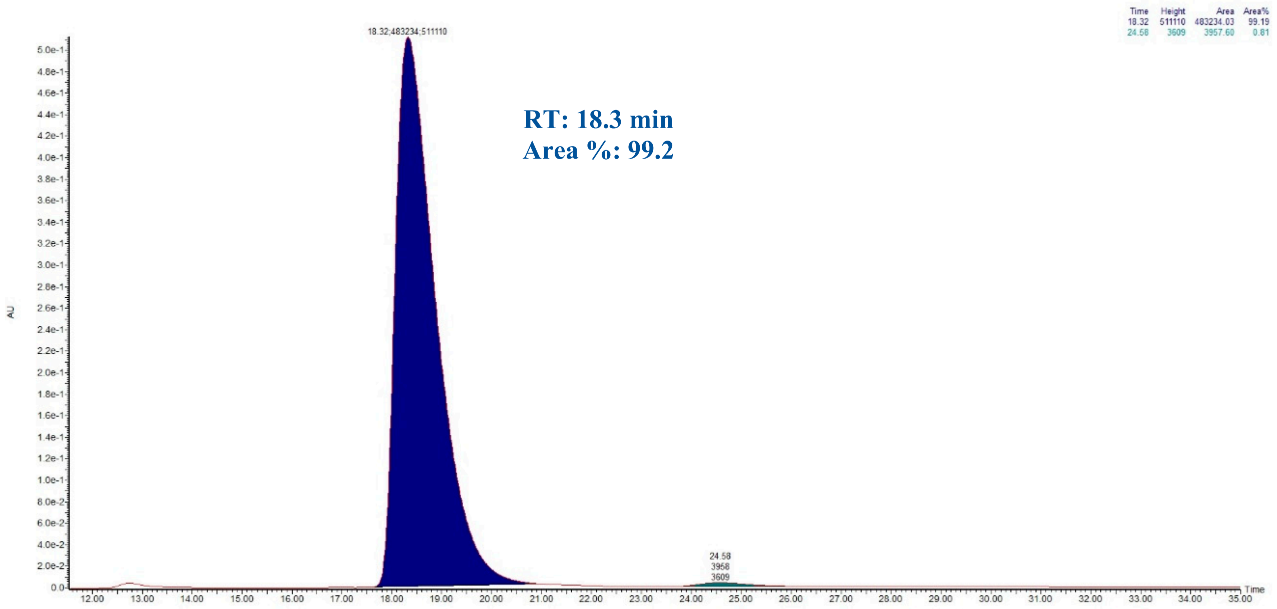

Figure S1 – chiral analytical HPLC: analysis of (+)-3-nitroatenolol

**Sample:**	NAT1 – Peak 1	**Flow:**	1 mL/min
**Column:**	5 AMY-Coat -5 µm Phenomenex	**Wavelenght:**	254/280 nm
**Dimension:**	250 x 4,6 mm	**Mobile Phase:**	n-hexane-ethanol 80:20

LC-MS Analysis - Peak 1 - (+)-3-nitroatenolol

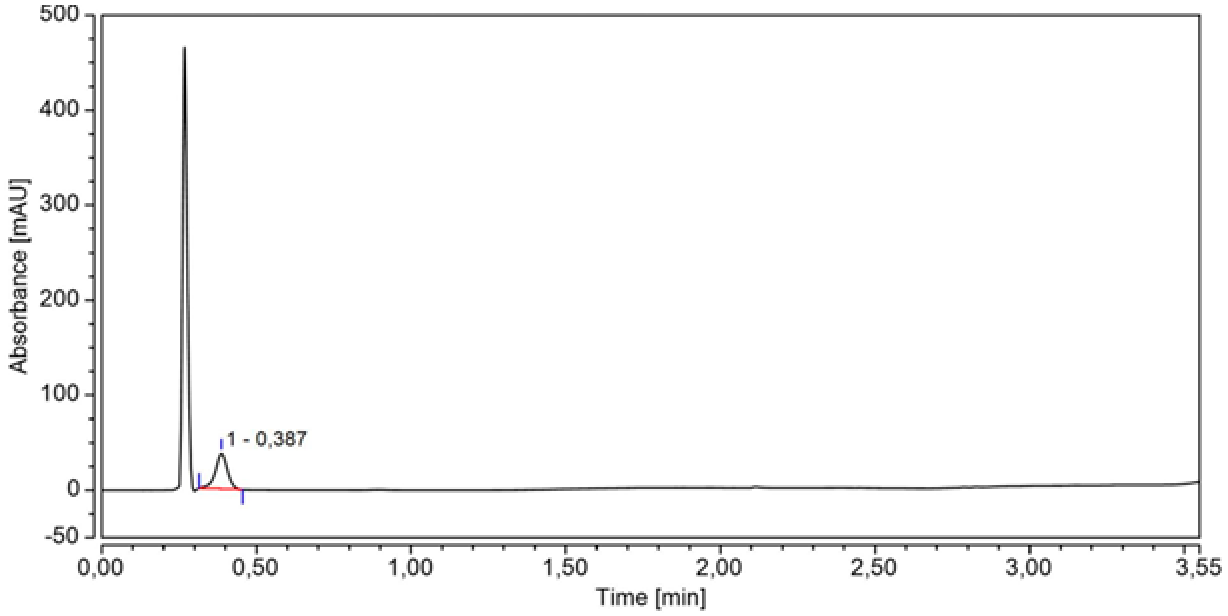

Peak Name	Retention Time min	Area mAU*min	Relative Area %
**1**	0.38	1.67	100

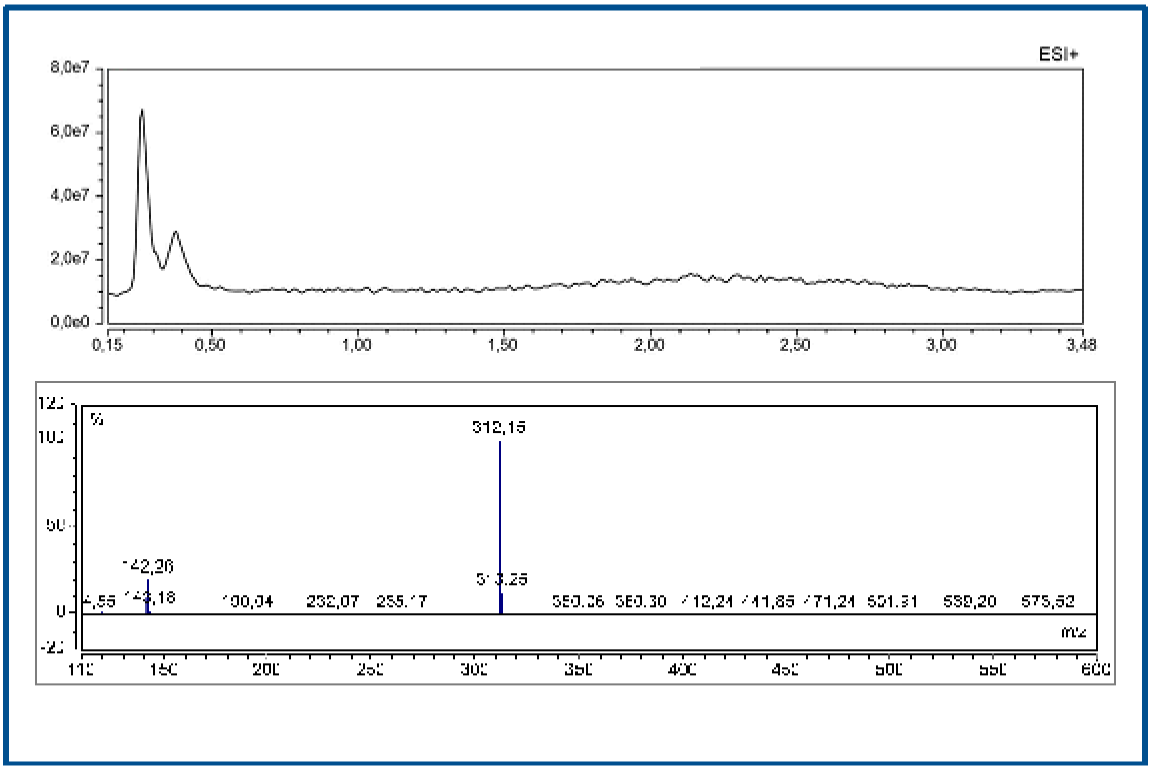

Peak 2 - *(-)*-3-nitroatenolol

Item	Specification	Result
**Appearance**	Yellow powder	Compliant
**Purity**	LC-MS purity >95%	Compliant
**Enantiomeric purity**	HPLC > 95%	Compliant
**Amount**	23.7 mg	

Stereochemical purity

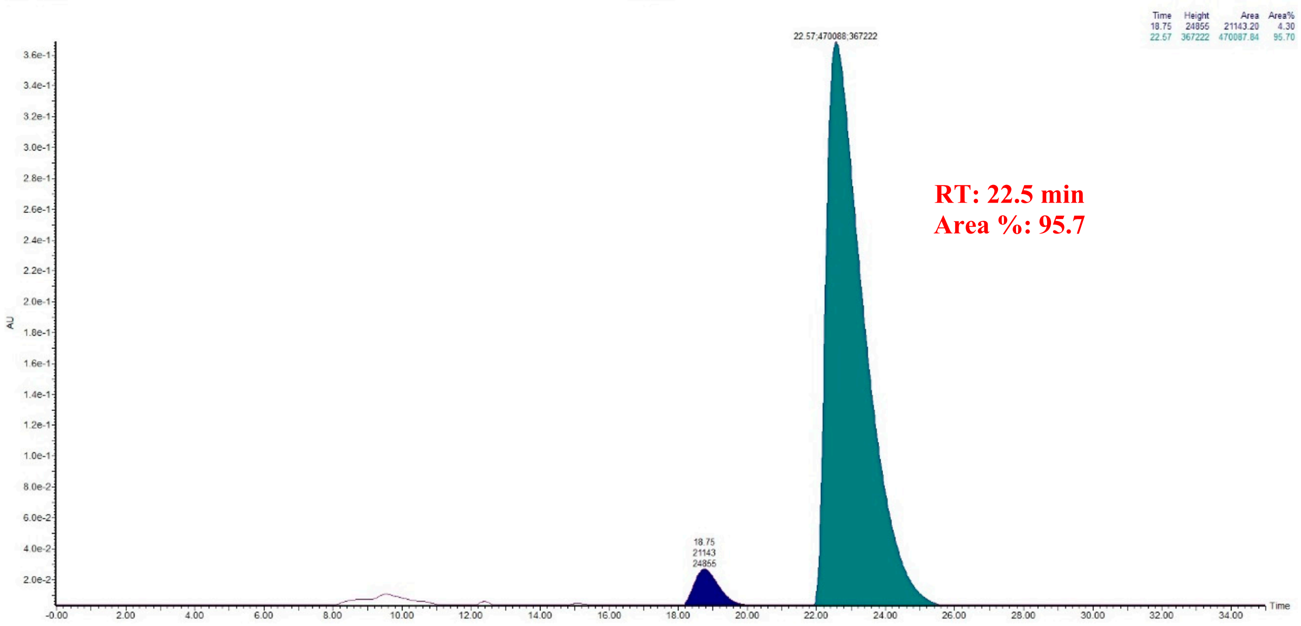

Figure S2 – chiral analytical HPLC: analysis of (-)-3-nitroatenolol.

**Sample:**	NAT1 – Peak 2	**Flow:**	1 mL/min
**Column:**	5 AMY-Coat -5 µm Phenomenex	**Wavelenght:**	254/280 nm
**Dimension:**	250 x 4,6 mm	**Mobile Phase:**	n-hexane-ethanol 80:20

LC-MS Analysis

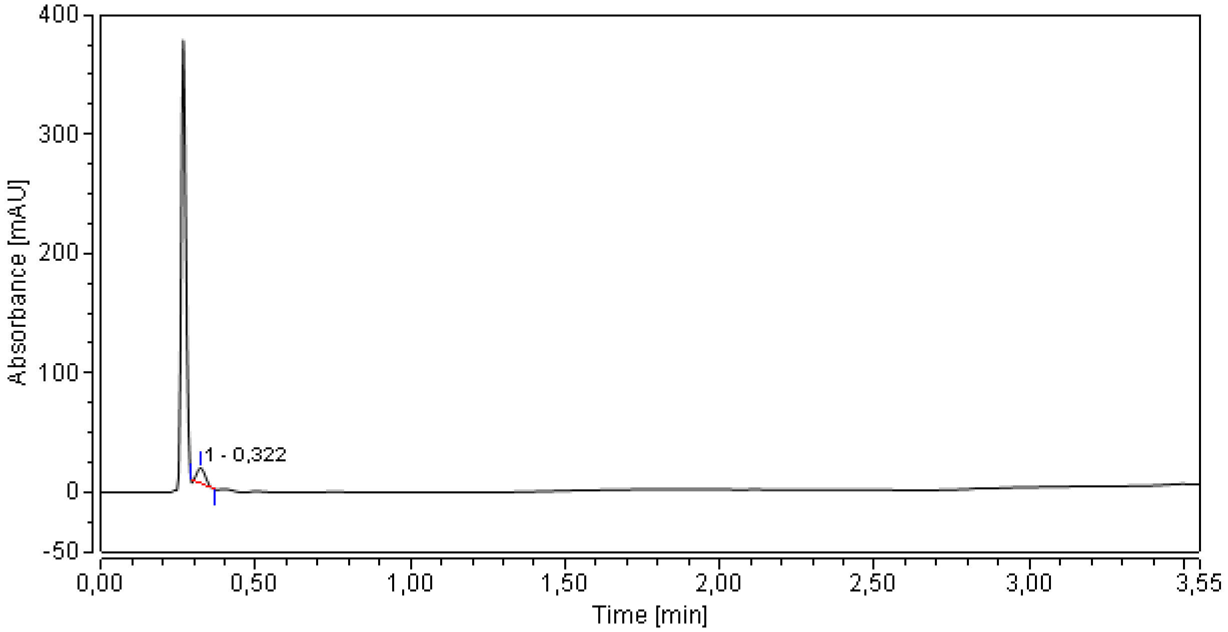

Peak Name	Retention Time min	Area mAU*min	Relative Area %
**1**	0.32	0.37	100

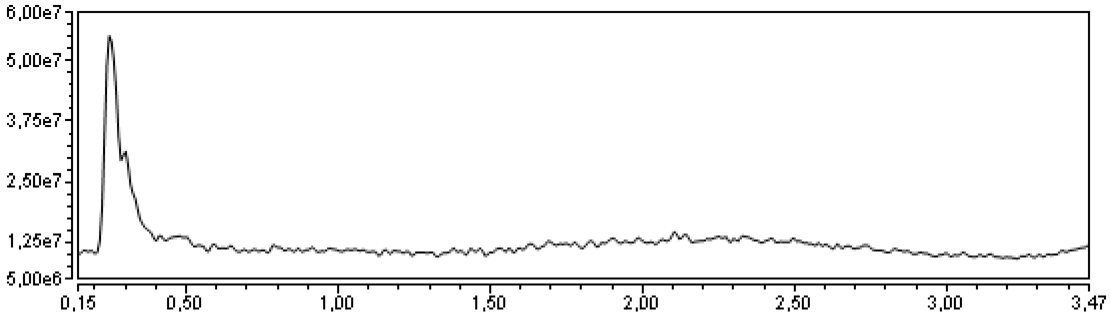

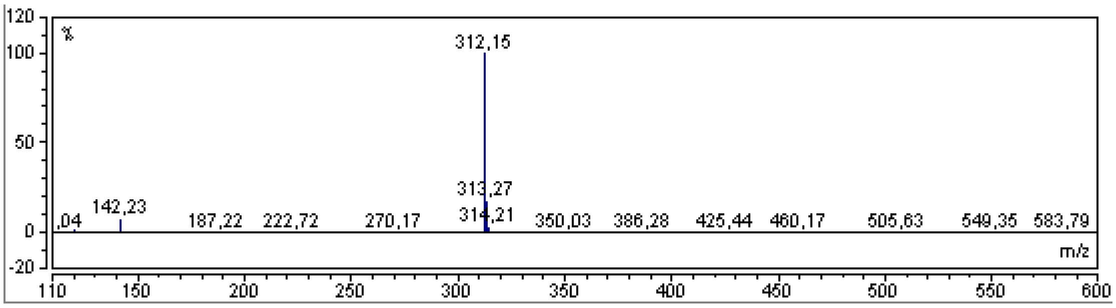

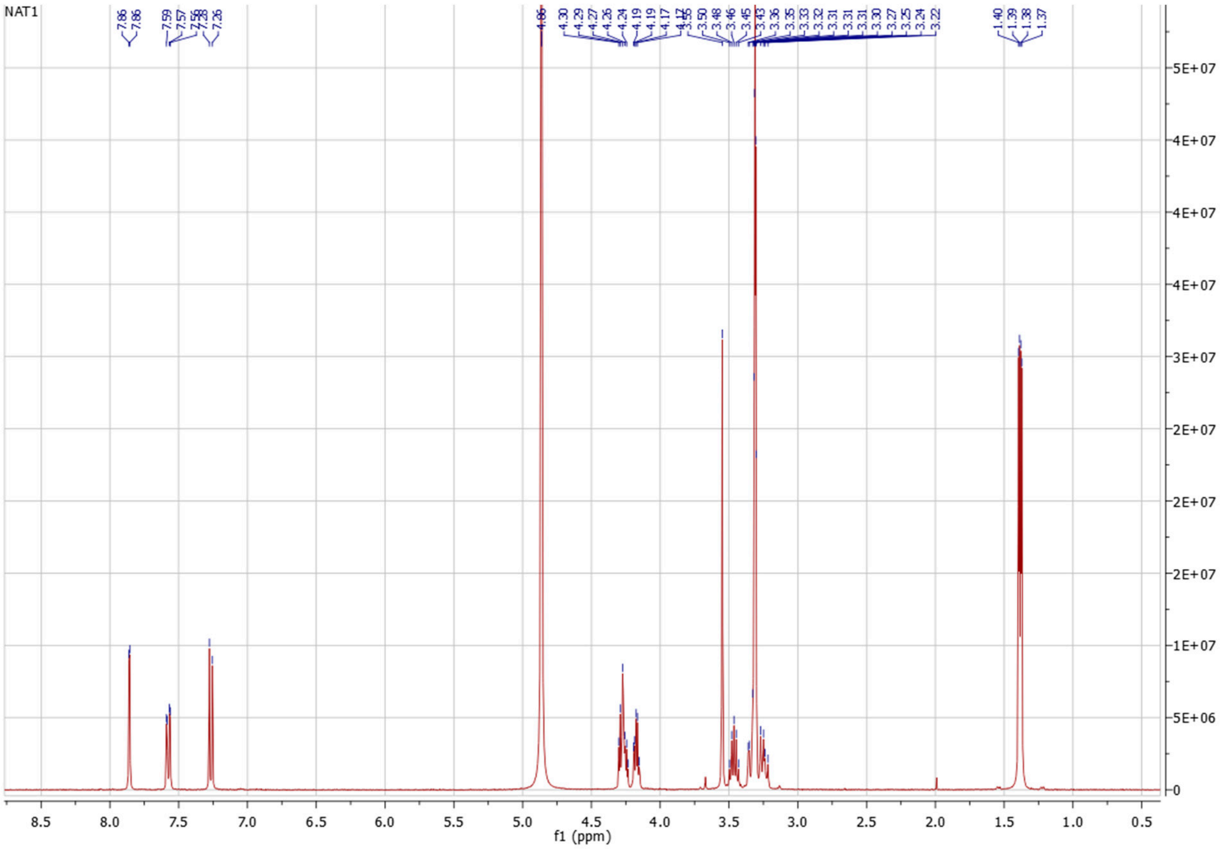

Figure S3 - ^{1}H-NMR of 3-nitroatenolol (CD_3OD)

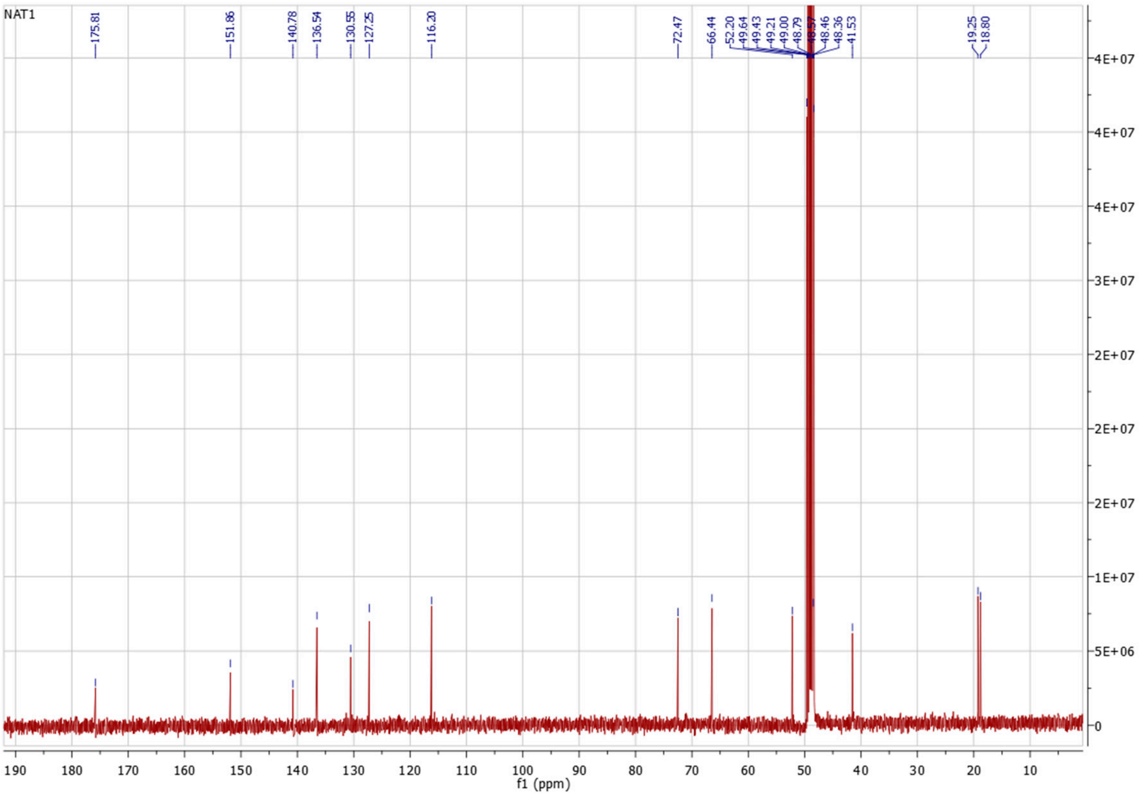

Figure S4 - ^{13}C-NMR of 3-nitroatenolol (CD_3OD)

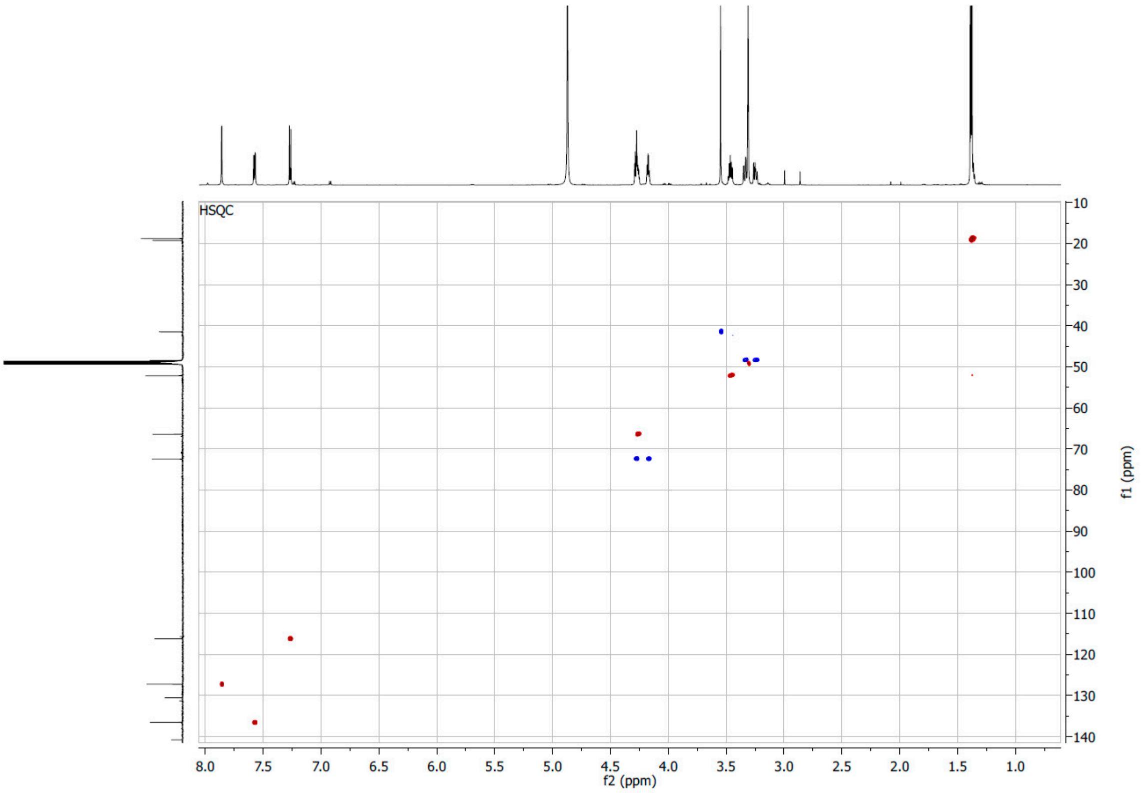

Figure S5 - HSQC of 3-nitroatenolol

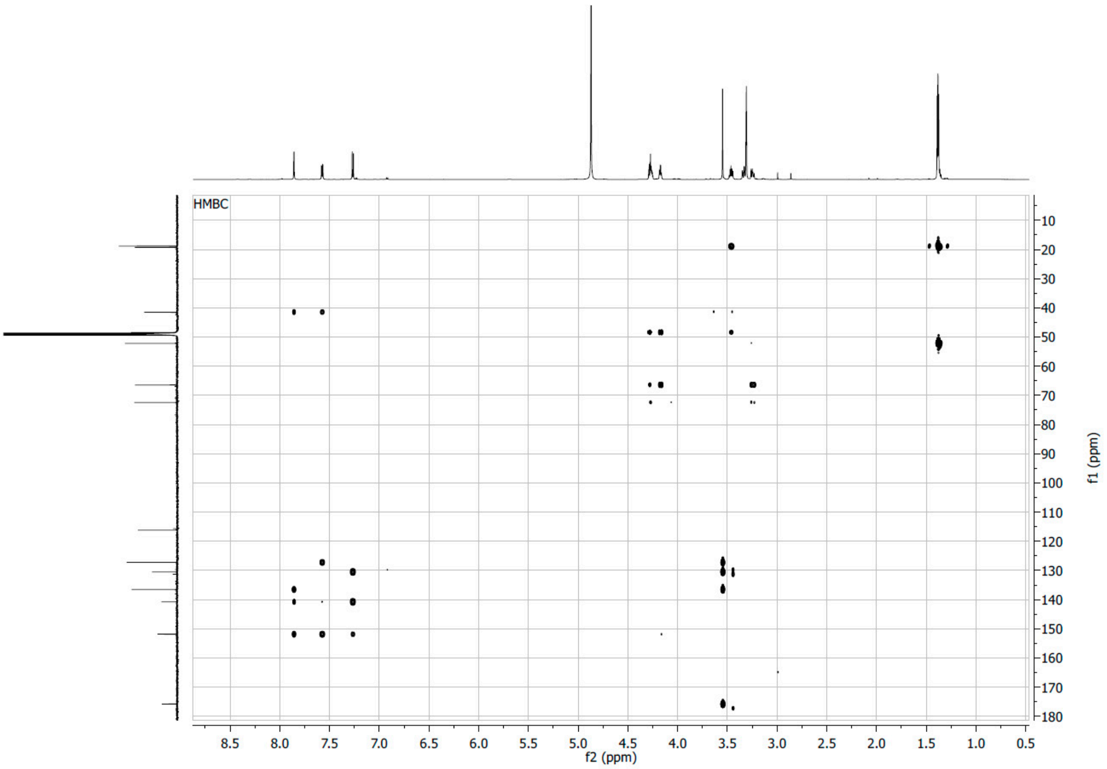

Figure S6 - HMBC of 3-nitroatenolol

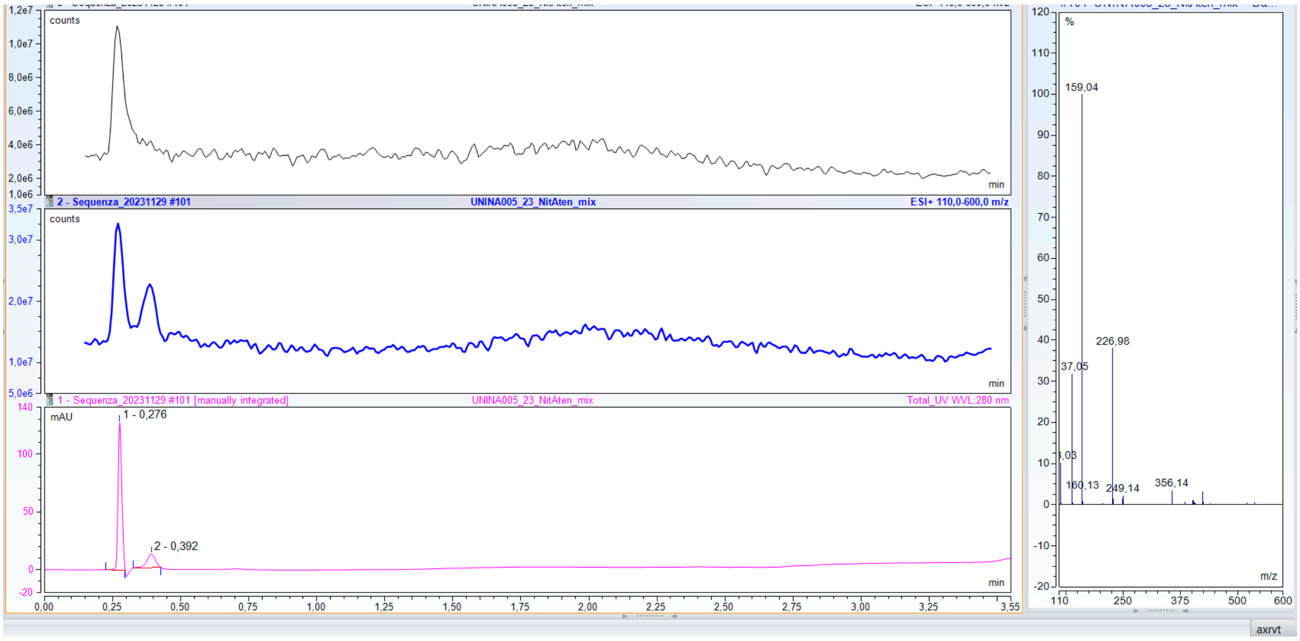

Figure S7 - LC-MS of (±)-3-nitroatenolol Pre-SCX. Mass spectrum in ESI^{+} (blue profile) matches with MW of compound. We detect in ESI^{-} (black profile) a peak corresponding to TFA (Dimer in negative mode: 226.9).

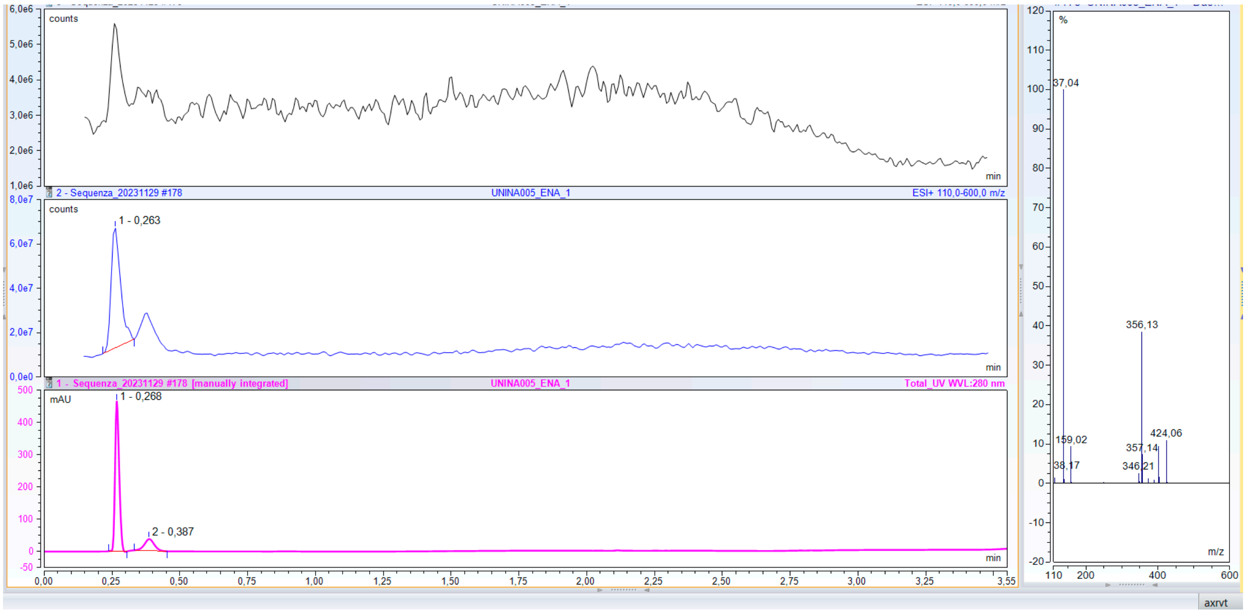

Figure S8 - LC-MS of (±)-3-nitroatenolol Post-SCX. In ESI^{-} (black profile) we observed only a peak corresponding to 3-nitroatenolol $[M+HCOOH]^{-}$

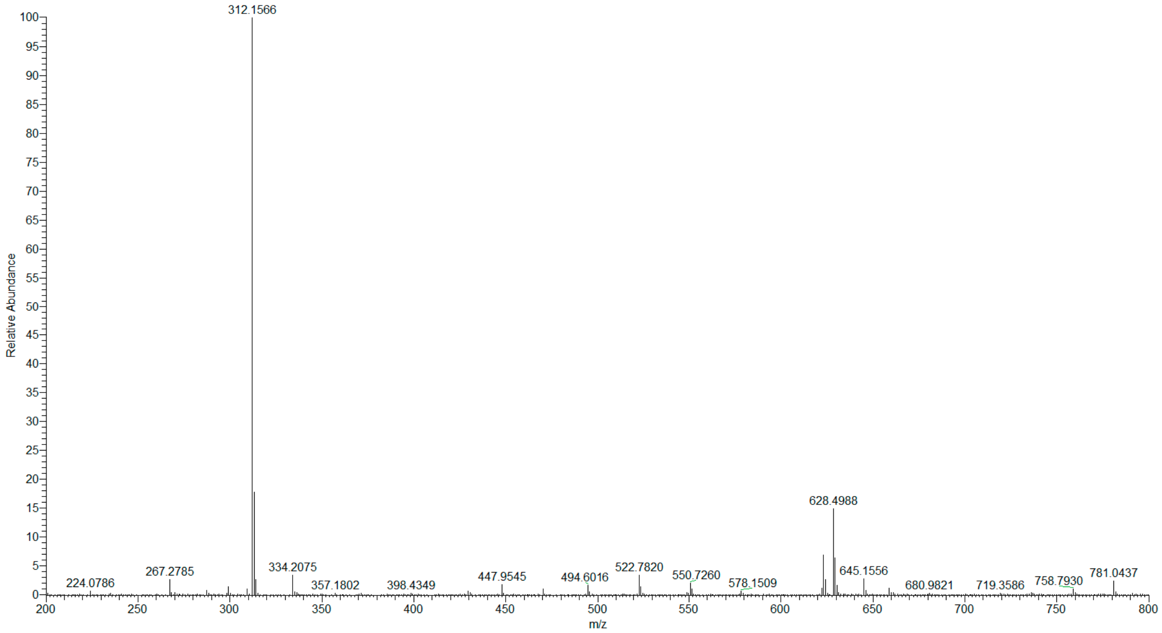

Figure S9 - HRESI-MS spectrum of (±)-3-nitroatenolol.

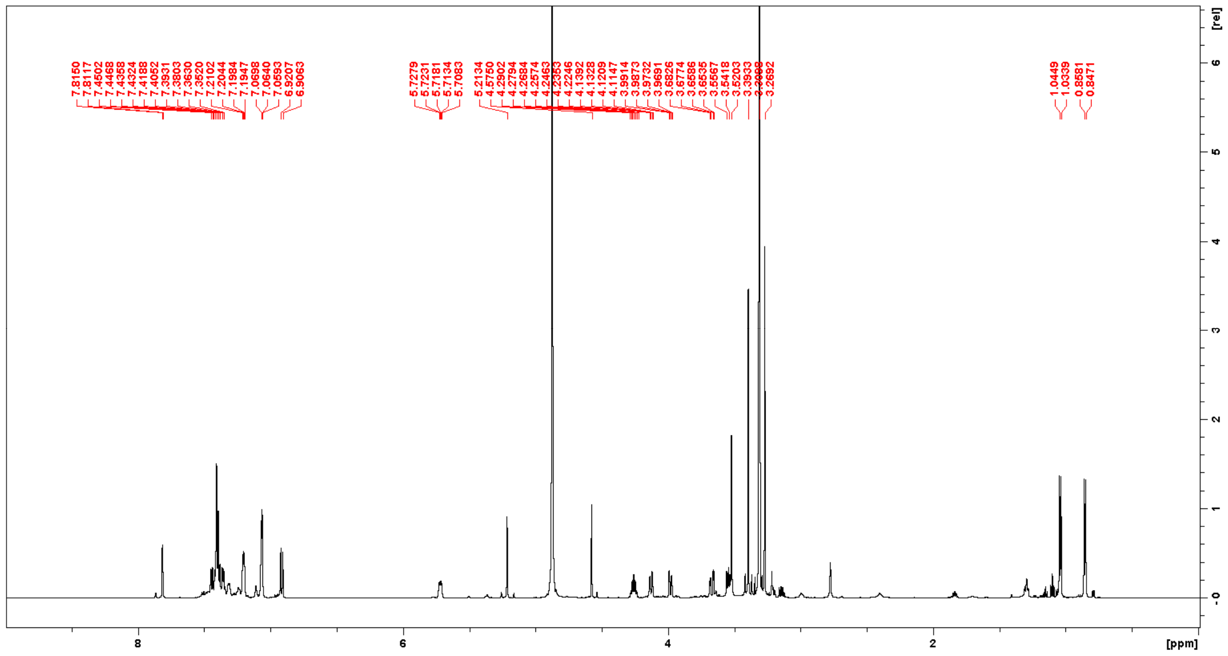

Figure S10 -^{1}H NMR spectrum in CD3OD of bis-(R)-MPA-(+)-3-nitroatenolol.

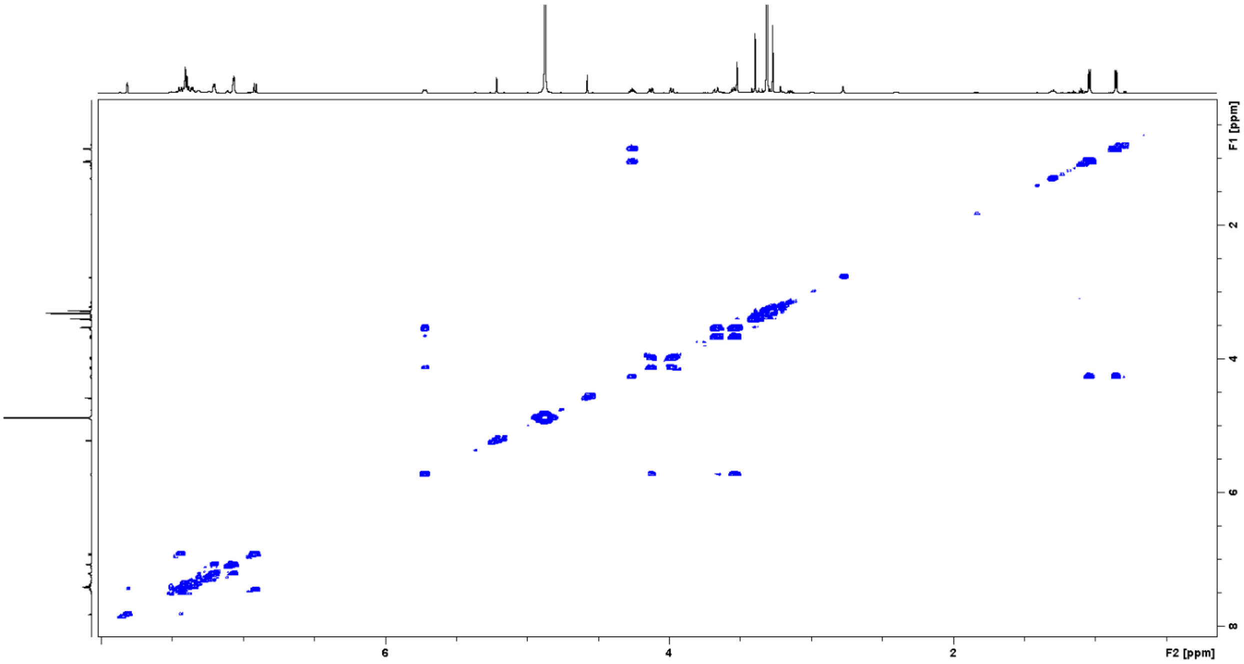

Figure S11 – COSY spectrum in CD_3OD of bis-(R)-MPA-(+)-3-nitroatenolol.

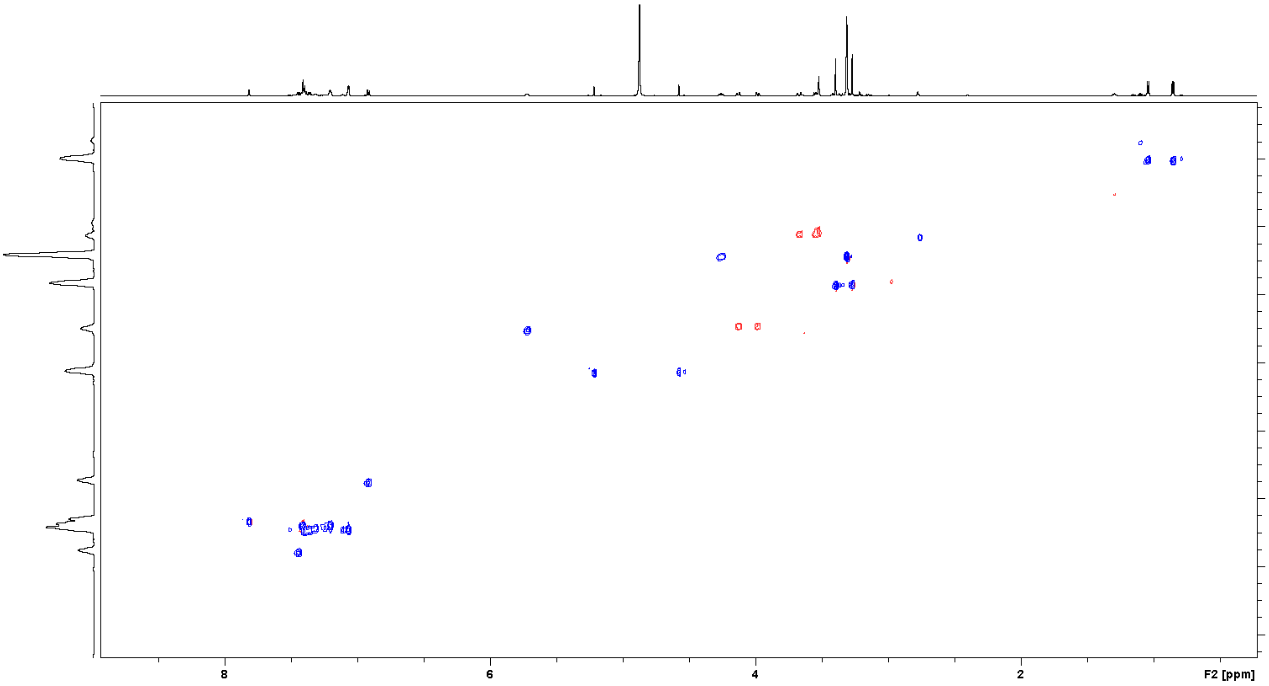

Figure S12 - HSQC spectrum in CD_3OD of bis-(R)-MPA-(+)-3-nitroatenolol.

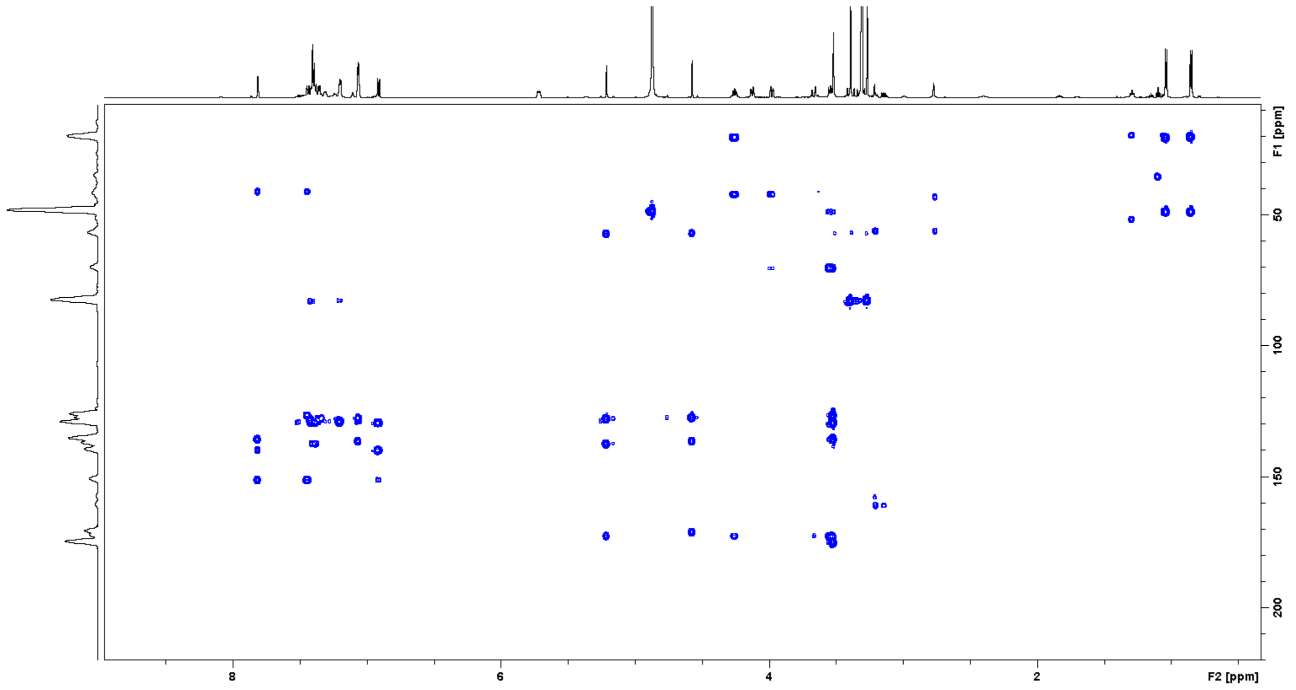

Figure S13- HMBC spectrum in CD_3OD of bis-(R)-MPA-(+)-3-nitroatenolol.

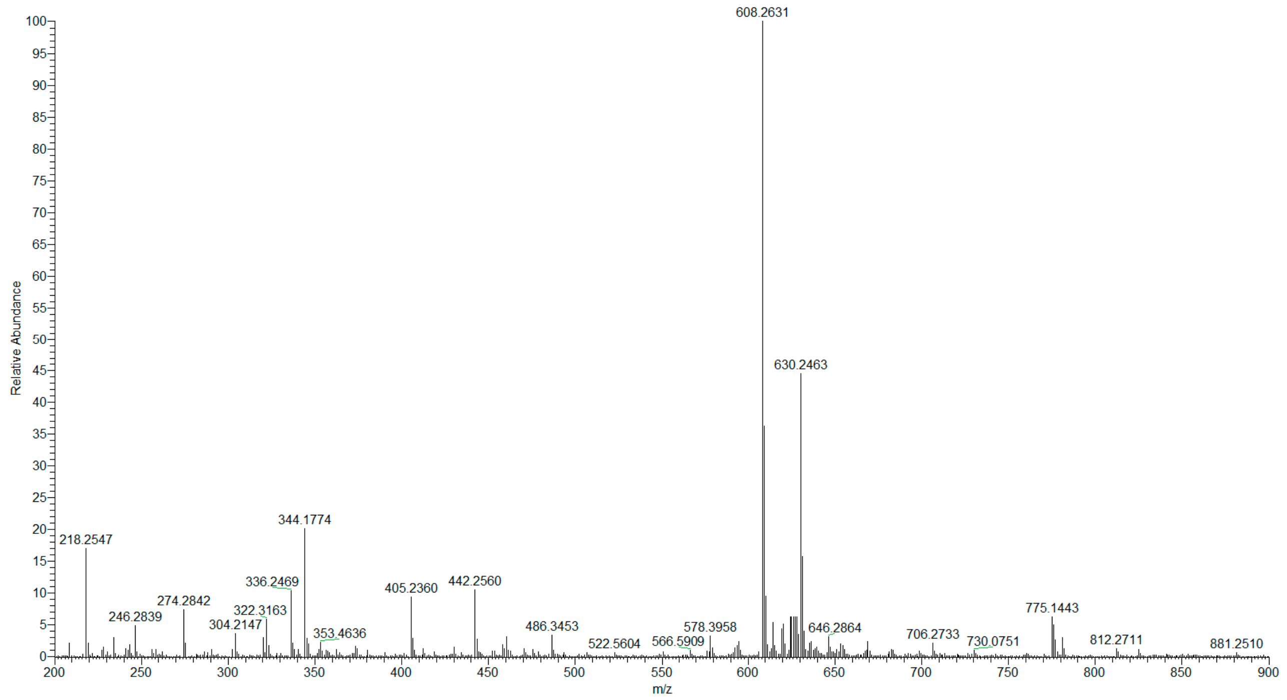

Figure S14 – HRESI-(+)-MS spectrum of bis-(R)-MPA-(+)-3-nitroatenolol.

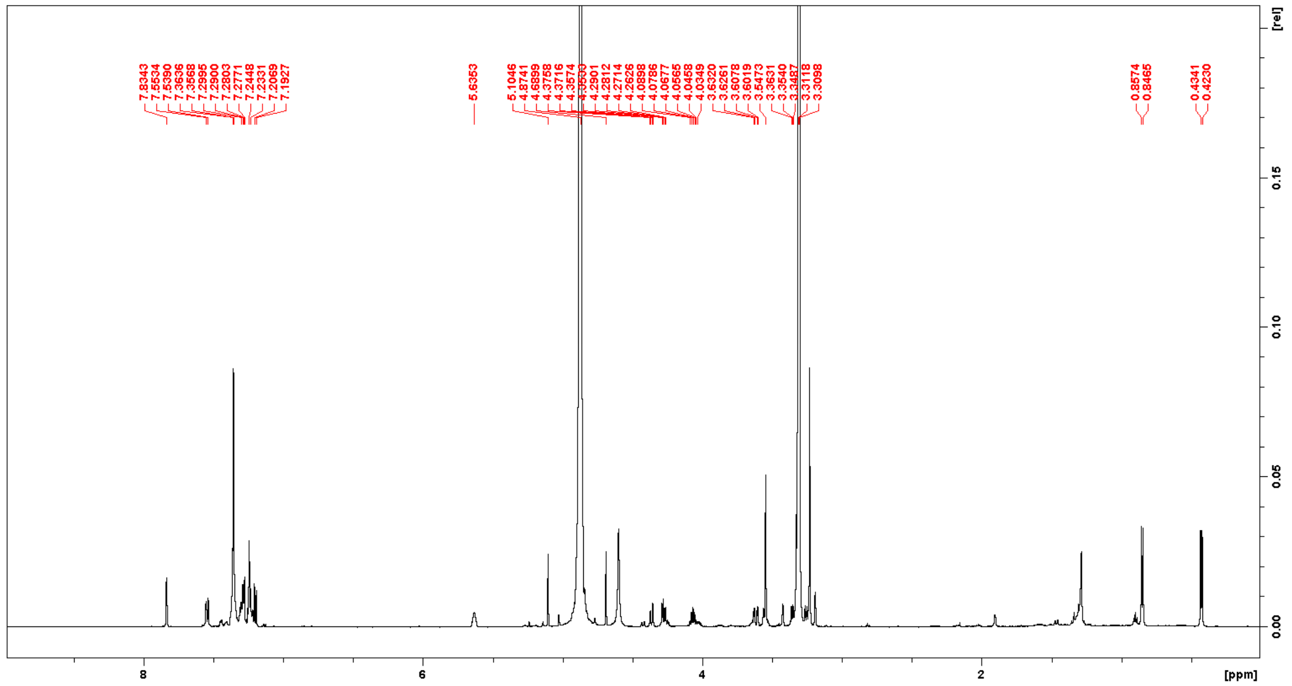

Figure S15- ^{1}H-NMR of bis-(S)-MPA-(+)-3-nitroatenolol.

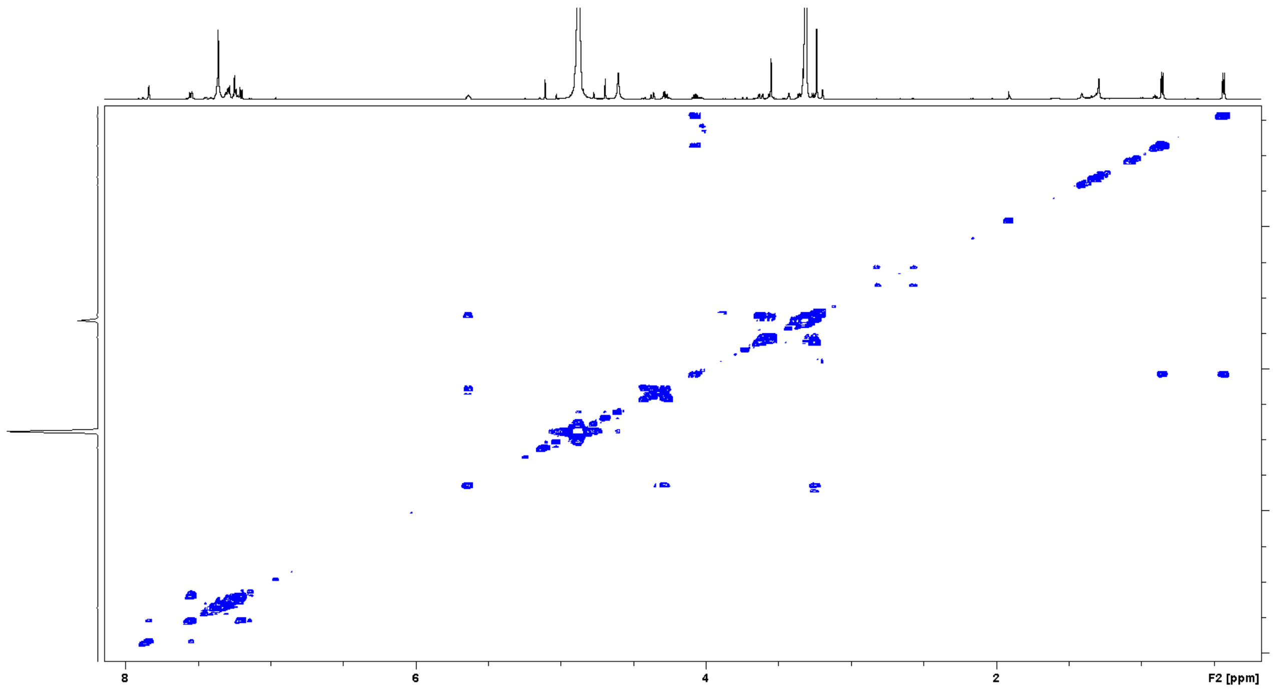

Figure S16 – COSY spectrum of bis-(S)-MPA-(+)-3-nitroatenolol.

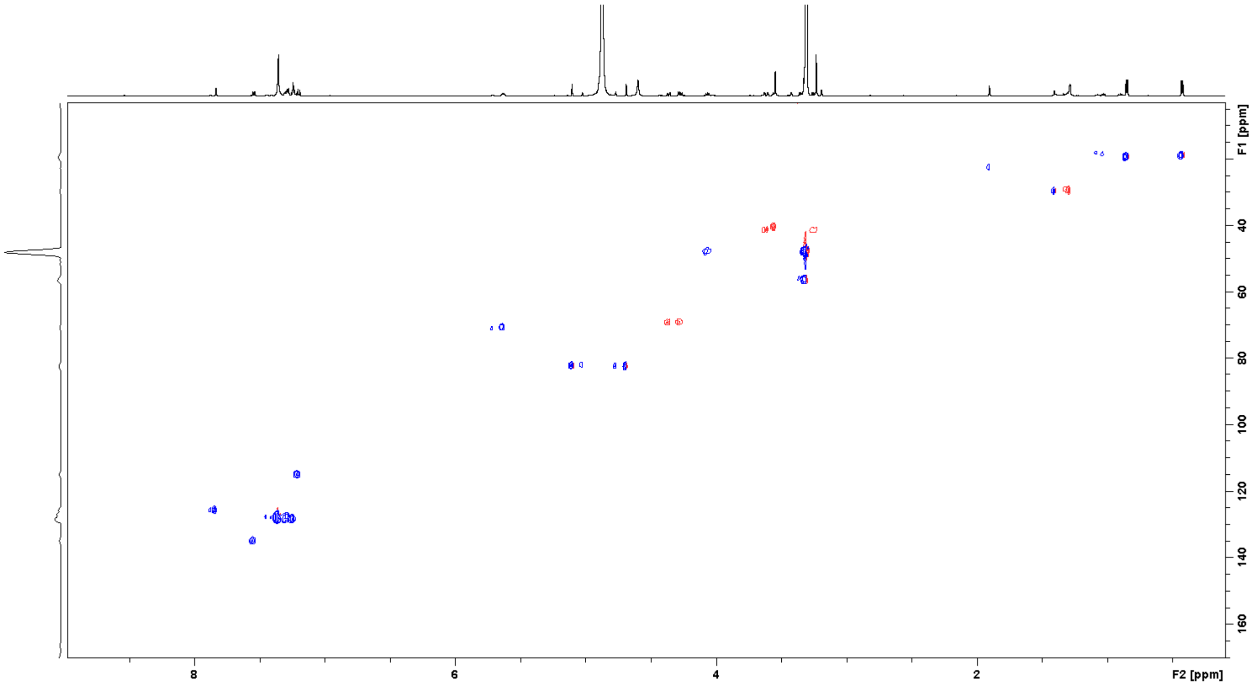

Figure S17 – HSQC spectrum of bis-(S)-MPA-(+)-3-nitroatenolol.

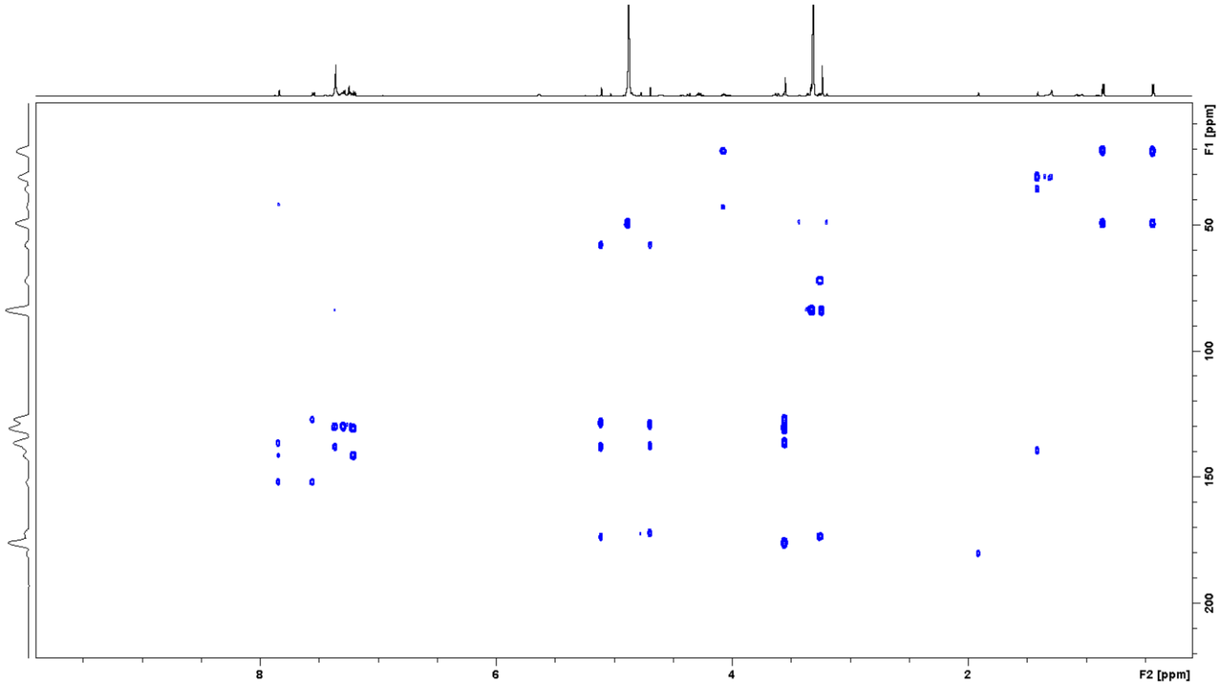

Figure S18 – HMBC spectrum of bis-(S)-MPA-(+)-3-nitroatenolol.

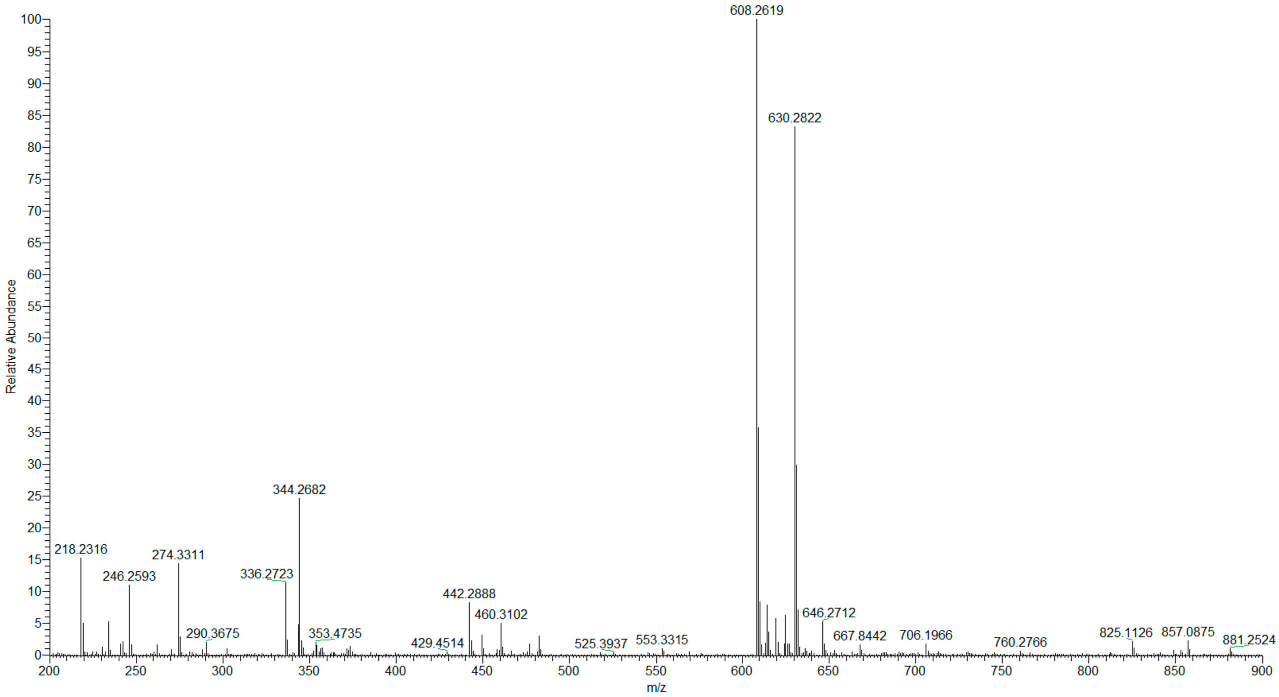

Figure S19 – HRESI-(+)-MS spectrum of bis-(S)-MPA-(+)-3-nitroatenolol.

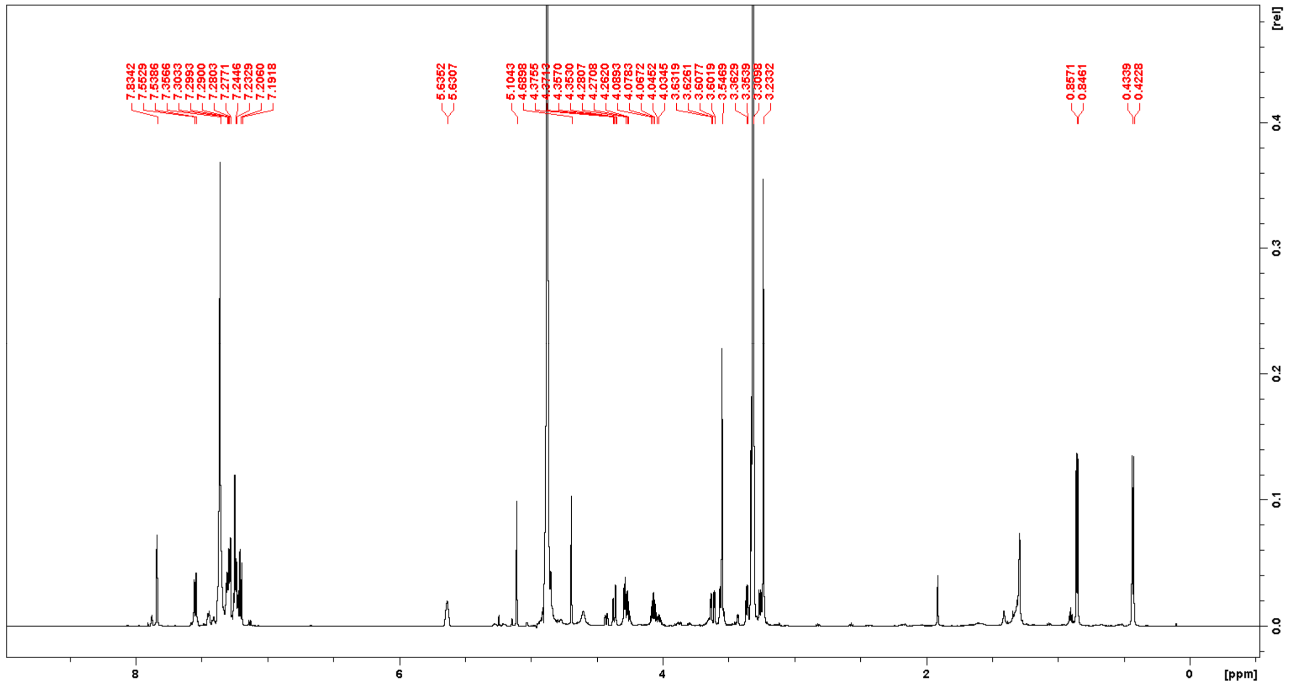

Figure S20 – NMR signals of bis-(R)-MPA derivative of (-)-3-nitroatenolol.

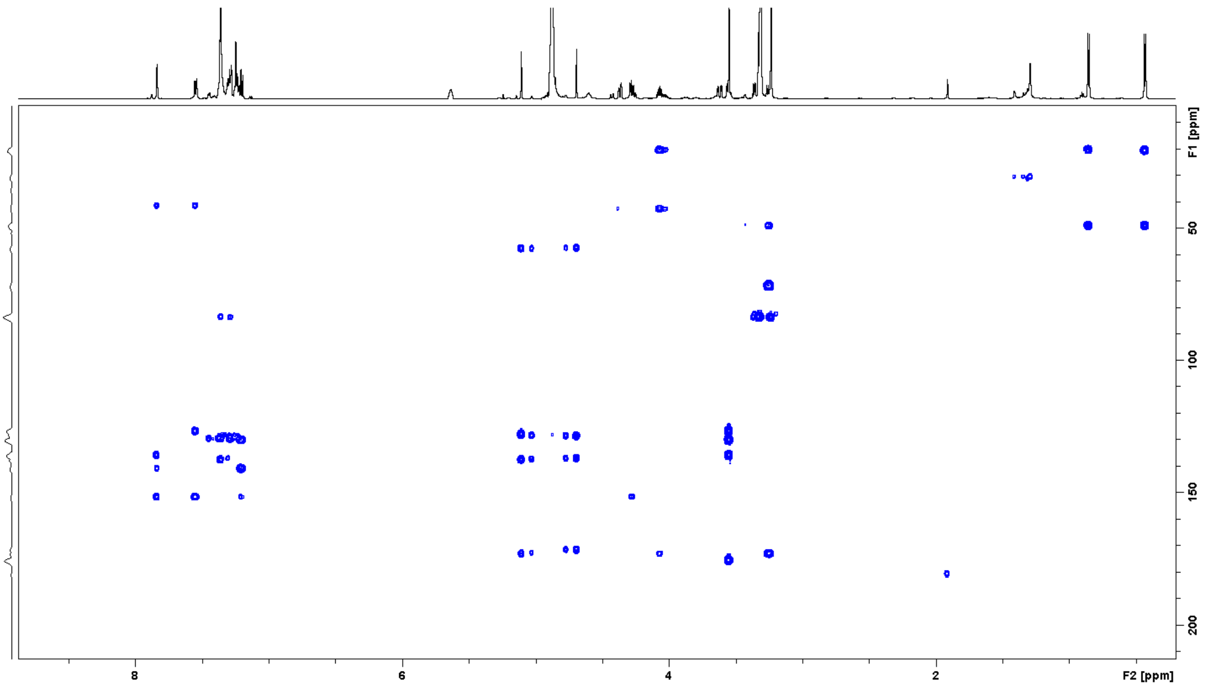

Figure S21 – HMBC spectrum of bis-(R)-MPA-(-)-3-nitroatenolol.

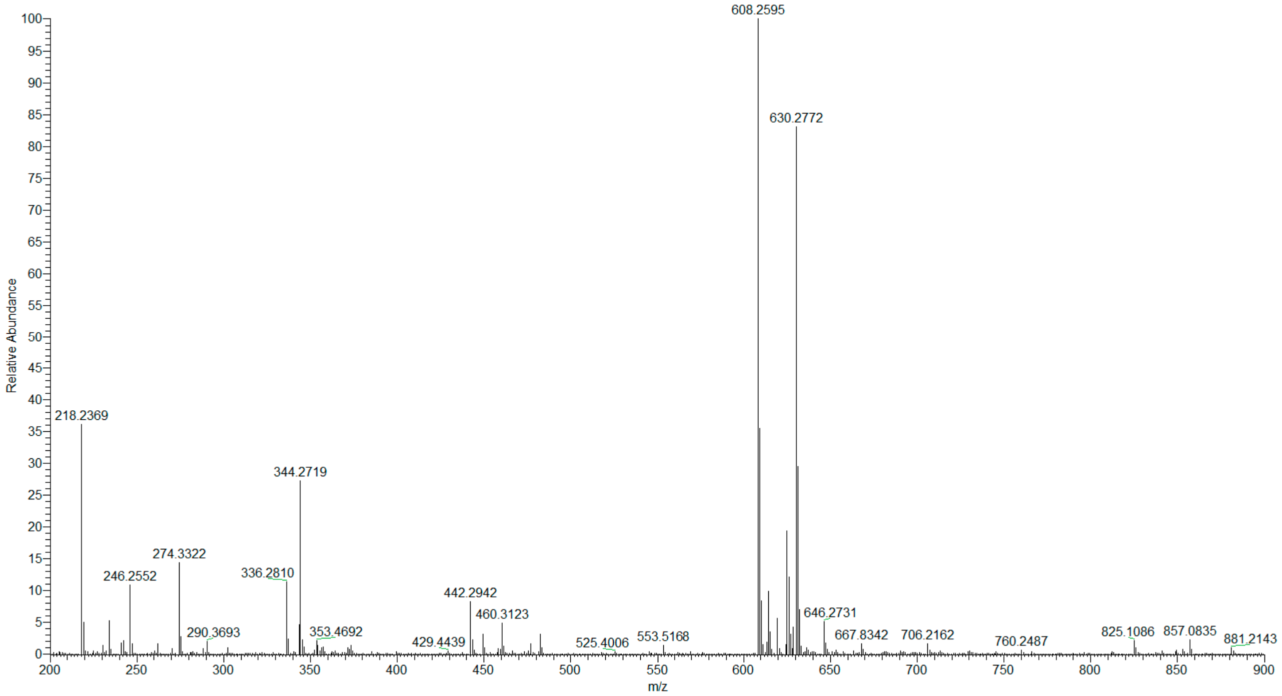

Figure S22 – HRESI-MS spectrum of bis-(R)-MPA-(-)-3-nitroatenolol.

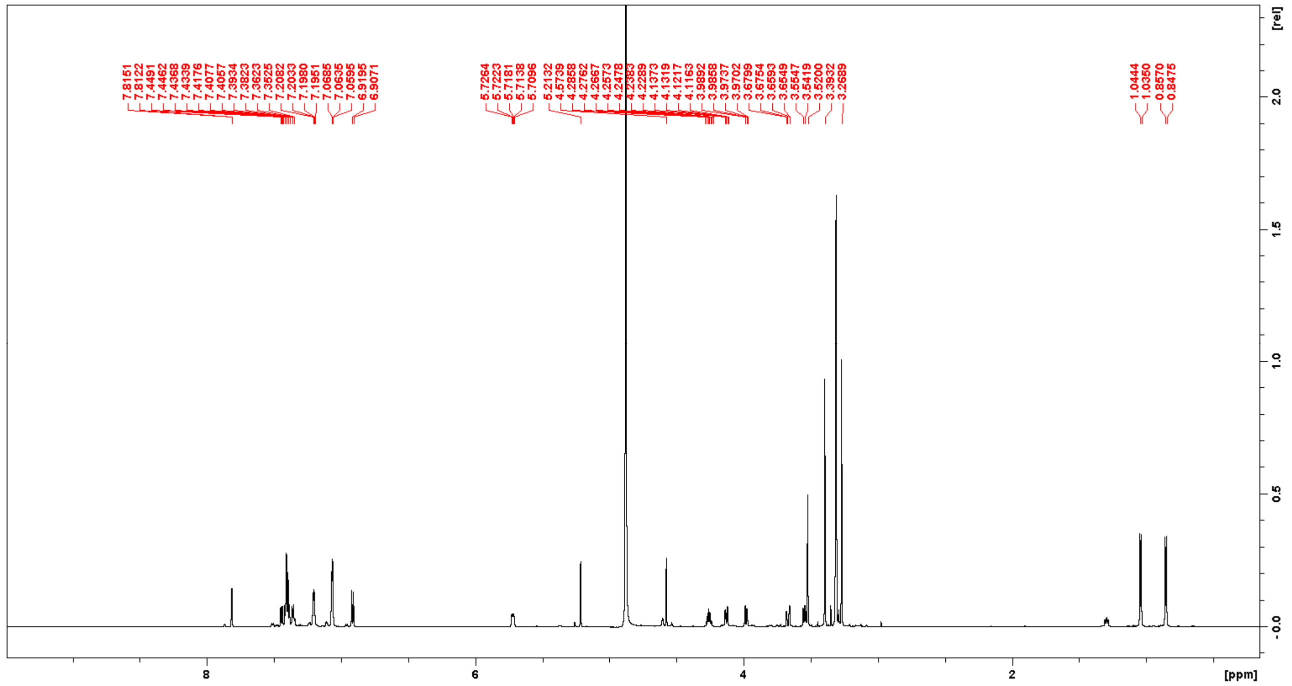

Figure S23 – NMR signals of bis-(S)-MPA derivative of (-)-3-nitroatenolol.

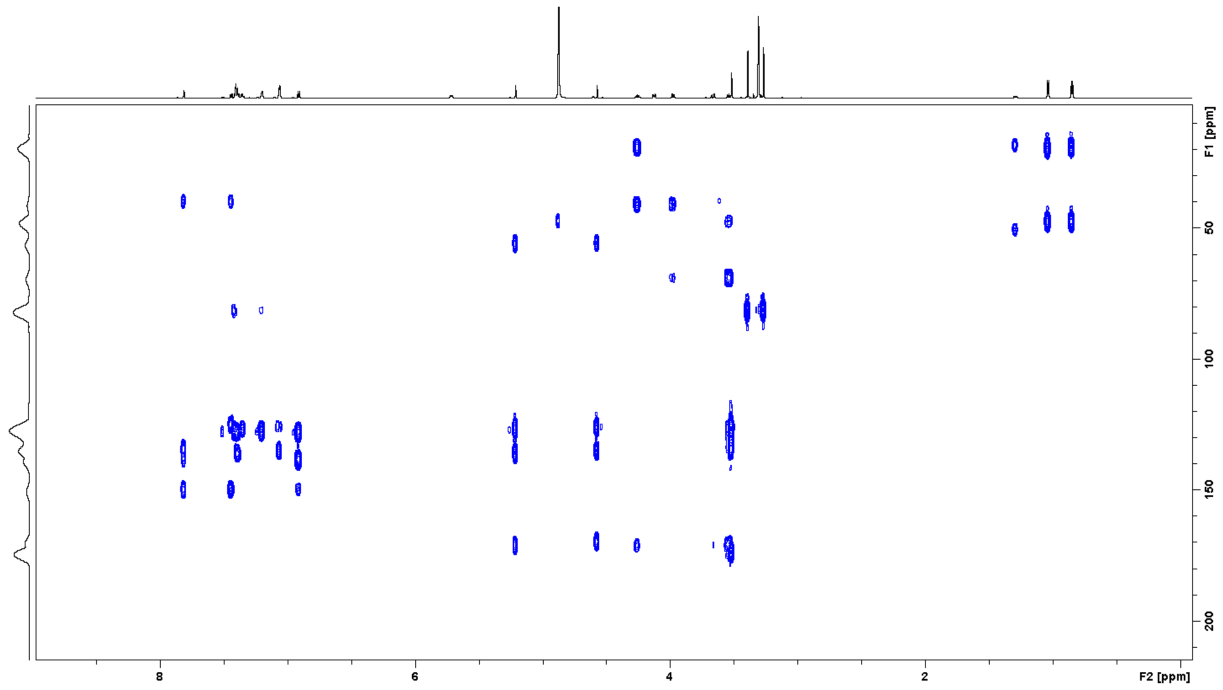

Figure S24 - HMBC of bis-(S)-MPA-(-)-3-nitroatenolol.

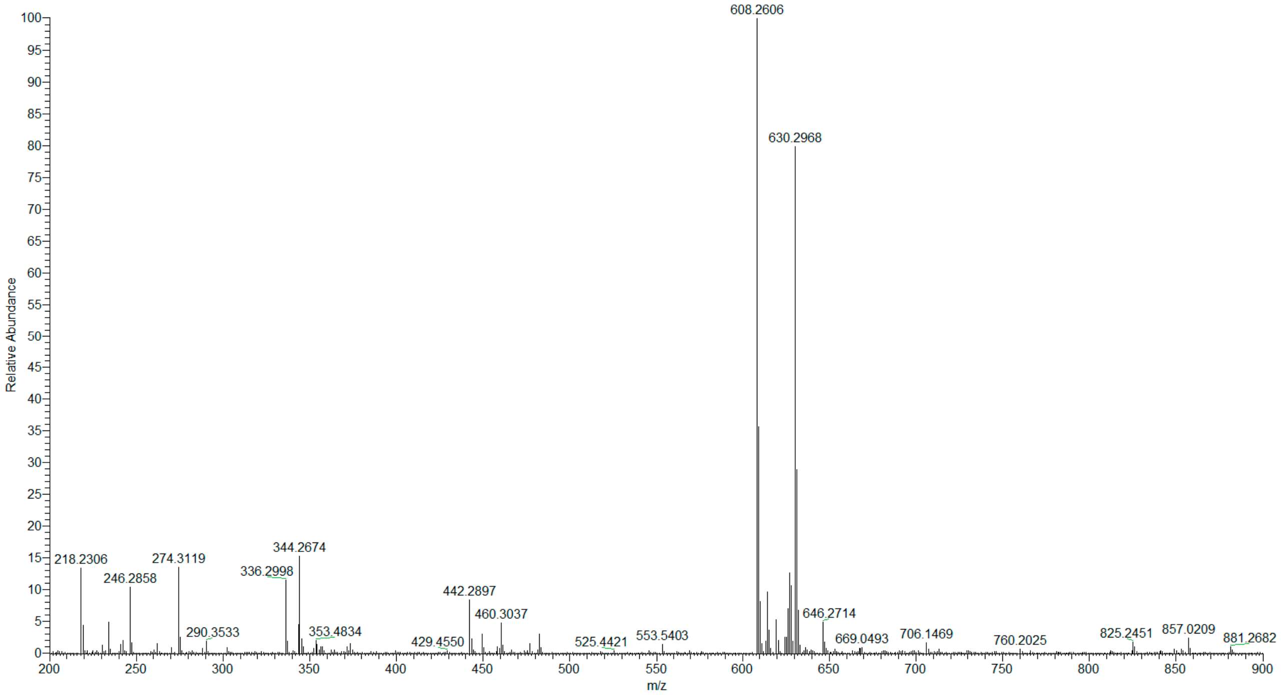

Figure S25 – HRESI-MS spectrum of bis-(S)-MPA-(-)-3-nitroatenolol.
